# Fast Penalized Generalized Estimating Equations for Large Longitudinal Functional Datasets

**Published:** 2025-06-25

**Authors:** Gabriel Loewinger, Alexander W. Levis, Erjia Cui, Francisco Pereira

**Affiliations:** 1 Machine Learning Core, National Institute of Mental Health; 2 Department of Statistics & Data Science, Carnegie Mellon University; 3 Department of Biostatistics and Health Data Science, University of Minnesota

**Keywords:** functional data analysis, longitudinal data analysis, calcium imaging, generalized estimating equations, one-step estimators

## Abstract

Longitudinal binary or count functional data are common in neuroscience, but are often too large to analyze with existing functional regression methods. We propose one-step penalized generalized estimating equations that supports continuous, count, or binary functional outcomes and is fast even when datasets have a large number of clusters and large cluster sizes. The method applies to both functional and scalar covariates, and the one-step estimation framework enables efficient smoothing parameter selection, bootstrapping, and joint confidence interval construction. Importantly, this semi-parametric approach yields coefficient confidence intervals that are provably valid asymptotically even under working correlation misspecification. By developing a general theory for adaptive one-step M-estimation, we prove that the coefficient estimates are asymptotically normal and as efficient as the fully-iterated estimator; we verify these theoretical properties in extensive simulations. Finally, we apply our method to a calcium imaging dataset published in *Nature*, and show that it reveals important timing effects obscured in previous non-functional analyses. In doing so, we demonstrate scaling to common neuroscience dataset sizes: the one-step estimator fits to a dataset with 150,000 (binary) functional outcomes, each observed at 120 functional domain points, in only ~ 13.5 minutes on a laptop without parallelization. We release our implementation in the fastFGEE package.

## Introduction

Neuroscience studies in animal models provide an invaluable tool to identify the neural mechanisms underpinning brain function and their relationship with psychiatric disorders. Researchers can estimate moment-by-moment associations between experimental covariates (e.g. behavior) and the activity of hundreds of neurons per animal, with widely-used in vivo brain recording techniques like calcium imaging (Grienberger et al., 2022) and *Neuropixels* (Jun et al., 2017). A neuroscientist might study brain-behavior associations on, for example, a learning task in which an animal learns to press a lever for a food reward. These tasks are often performed over hundreds of experimental replicates called “trials” (longitudinal observations akin to “patient visits”). Each trial might be defined as a five second interval starting at extension of the lever and ending with delivery of the food reward. To test whether, for example, mean neural activity is higher on trials when animals press the lever, a common strategy is to analyze scalar summaries of each trial’s neuronal firing activity. For instance, analysts might calculate a firing rate of neuron i on trial j by averaging the response, $Y_{i,j}(s)\in \{0,1\}$, across within-trial timepoints indexed by $s:{\overset{‾}{Y}}_{i,j}=\frac{1}{\left|𝒮\right|}\sum_{s\in 𝒮}Y_{i,j}(s)$, where 𝒮⊂[0,5] denotes a grid of timepoints at which the outcome is observed. One might then test whether $E\left[{\overset{‾}{Y}}_{i,j}|X_{i,j}=1\right]-E\left[{\overset{‾}{Y}}_{i,j}|X_{i,j}=0\right]\neq 0$, where $X_{i,j}$ is an indicator that the animal on which neuron i was recorded pressed the lever on trial j. This analysis approach is parsimonious but discards important temporal information by summarizing across trial timepoints, s.

Alternatively, the neural response of each five second trial can be conceptualized as a functional outcome, with within-trial timepoints, s, representing locations along the functional domain. This allows one to apply functional data analysis (FDA, Crainiceanu et al. (2024)) techniques to test how brain–behavior relationships evolve within and across trials (Loewinger et al., 2025). FDA offers a flexible framework to analyze a wide range of neuroscience studies, as it allows both the responses and the covariates (e.g. behavior) to be functional. The size and complexity of our dataset, however, require specialized FDA methods. First, analyses must account for the longitudinal structure, since each neuron’s activity is collected across many trials. Second, the large number of clusters (i.e. neurons) and large cluster sizes (i.e. number of trials recorded for a given neuron), often in the hundreds or thousands, make many longitudinal FDA methods for binary or count functional outcomes computationally impractical.

To conduct inference in longitudinal FDA with large datasets, we propose a one-step estimator for functional generalized estimating equations (fGEE). Procedurally, we first fit a function-on-scalar regression with a working independence correlation structure to obtain a consistent but potentially inefficient initial estimate of the functional coefficients. We then update the initial estimate with one Newton-Raphson update step, derived from an estimating equation that models intra-cluster correlation. This approach can scale to large datasets and has desirable statistical properties. The initial estimate can be formed quickly because it ignores correlation; using only ‘one step’ in the update is fast because it greatly reduces the number of times potentially large working covariance matrices are inverted. Importantly, our approach still captures much of the statistical efficiency afforded by modeling intra-cluster correlation in longitudinal and/or functional directions with a fully-iterated fGEE. In fact, we prove the one-step fGEE is asymptotically as efficient as the fully-iterated version.

We provide an implementation that supports functional data observed on regular, irregular, dense and sparse grids with functional and/or scalar covariates (see Appendix A.4 for implementation details and Section 7 for links to our Github repos). We further propose adoption of working correlation structures that allow for use of algorithms to efficiently construct and invert large working covariance matrices. We also propose fast strategies for smoothing parameter tuning, cluster bootstrapping, and joint confidence interval construction. We review the literature in Section 1, present our estimator in Section 2, provide theoretical results in Section 3, simulations in Section 4, and a data application in Section 5.

## 1 Related Literature

We focus on the longitudinal function-on-scalar regression literature, where a wide range of conditional and marginal methods have been proposed (Eckardt et al., 2024; Sergazinov et al., 2023; Zhu et al., 2019; Scheipl et al., 2016; Shou et al., 2015; Brockhaus et al., 2015; Scheipl et al., 2015; Zipunnikov et al., 2014; Greven et al., 2011; Morris and Carroll, 2006; Guo, 2002).

Functional mixed models are a versatile conditional strategy for longitudinal FDA. For non-Gaussian functional outcomes, many existing approaches do not scale well to large cluster sizes or cluster numbers; see discussion and comparison in Cui et al. (2022). Cui et al. (2022); Loewinger et al. (2025); Zhou et al. (2025) proposed a fast functional mixed models approach based on univariate mixed models fits at each functional domain point. These rely on a cluster bootstrap for inference, however, which can be slow for large datasets. Moreover, for non-Gaussian outcomes these approaches yield coefficient estimates that are only interpretable as conditional on the random effects. In many applications, estimates with marginal interpretations are desirable.

Functional GEE and Quadratic Inference Functions (QIF) are marginal methods for longitudinal function-on-scalar regression. Qu and Li (2005) and Guha Niyogi and Zhong (2025) proposed QIF-based methods applicable to FDA, but, to the best of our understanding, these works focused on a single observation of a functional outcome per subject. Chen et al. (2013) proposed a penalized GEE for longitudinal FDA that serves as part of the inspiration for our work. The method, however, requires inverting an $n_{i}L\times n_{i}L$ matrix at each step of model fitting, where $n_{i}$ is the size of cluster i, and L is the number of points in the functional domain. Li et al. (2022) proposed a marginal estimator for continuous outcomes, but as we show in simulations, it does not scale to large cluster sizes, and has not been extended to binary or count outcomes. Taken together, marginal approaches for longitudinal functional regression with binary or count outcomes do not scale well, thereby limiting their widespread adoption.

Finally, there is a rich literature on penalized longitudinal marginal models for high dimensional covariates with sparsity; see Xia and Shojaie (2024) and references therein. However, we focus on functional data with low dimensional covariates where we do not encourage sparsity.

## 2 Methods

We begin by introducing notation, adopting that used in Li et al. (2022) where possible. We suppose that we observe the functional outcome $Y_{i,j}(s)$ at point s for cluster i∈[N]≔{1,…,N}, at longitudinal observation (e.g. trial or visit) $j\in \left[n_{i}\right]$. We express grids as regular (i.e. $n_{i}=n_{i}(s)\;\forall s\in \left\{s_{1},\ldots ,s_{L}\right\}$) and evenly spaced for ease of notation, but our methods also apply to irregular and unevenly spaced grids. We denote $Y_{i}(s)\in R^{n_{i}}$ as the functional outcome vector at point s for cluster i, concatenating all observations $Y_{i,j}(s)$ for $j\in \left[n_{i}\right]$, and write $Y_{i}={\left[Y_{i}{\left(s_{1}\right)}^{T},\ldots ,Y_{i}{\left(s_{L}\right)}^{T}\right]}^{T}\in R^{n_{i}L}$. We denote covariate vector $X_{i,j}\in R^{q}$ for cluster i on observation j, and $X_{i}={\left[X_{i,1},\ldots ,X_{i,n_{i}}\right]}^{T}\in R^{n_{i}\times q}$. We write covariates as scalar for ease of notation, though our method and theory applies to functional covariates.

### 2.1 Functional Generalized Estimating Equations

We consider the marginal function-on-scalar regression

(1) $$g\left(E\left[Y_{i,j}(s)|X_{i,j}\right]\right)=\eta_{i,j}(s),\;\eta_{i,j}(s)=\beta_{0}(s)+\sum_{r=1}^{q}X_{i,j,r}\beta_{r}(s),\;\text{Cov}\left(Y_{i}|X_{i}\right)=V_{i}^{*}$$

where g is a link function and $\beta_{r}(\cdot )$ is a (smooth) coefficient function for covariate r∈[q]. We let $\mu_{i,j}(s)=g^{-1}\left(\eta_{i,j}(s)\right)$ denote the mean function. We now discuss estimation of $\mu_{i,j}(s)$ with spline basis expansions of the $\beta_{r}(\cdot )$, although our methods can be used for other basis functions. For example, denoting $B(s)={\left[B_{1}(s),\ldots ,B_{m}(s)\right]}^{T}\in R^{m}$ as a set of m B-spline basis functions, we can represent the functional coefficients $\beta_{r}(s)=\sum_{d=1}^{m}\theta_{r,d}B_{d}(s)$. We denote $\theta_{r}={\left[\theta_{r,1},\ldots ,\theta_{r,m}\right]}^{T}\in R^{m}$ as an unknown parameter vector associated with covariate r, $B={\left[B\left(s_{1}\right),\ldots ,B\left(s_{L}\right)\right]}^{T}\in R^{L\times m}$, and the linear predictor for a full observation of the functional outcome as $\eta_{i,j}={\left[\eta_{i,j}\left(s_{1}\right),\ldots ,\eta_{i,j}\left(s_{L}\right)\right]}^{T}=B\theta_{0}+\sum_{r=1}^{q}X_{i,j,r}B\theta_{r}$. We further define $X_{i,j}=\left[B,X_{i,j,1}B,\ldots ,X_{i,j,q}B\right]\in R^{L\times p}$, where p=m(1+q). We then have that $\eta_{i,j}=X_{i,j}\theta$, where $\theta ={\left[\theta_{0}^{T},\theta_{1}^{T},\ldots ,\theta_{q}^{T}\right]}^{T}\in R^{p}$. Thus, we can estimate the functional coefficient vector, $\beta_{r}={\left[\beta_{r}\left(s_{1}\right),\ldots ,\beta_{r}\left(s_{L}\right)\right]}^{T}\in R^{L}$, by estimating θ and calculating ${\hat{\beta }}_{r}=B{\hat{\theta }}_{r}$.

We semi-parametrically estimate the θ with the penalized spline-based fGEE proposed in Chen et al. (2013). This assumes no likelihood and, if $\mu_{i,j}(s)$ is correctly specified, yields valid inference for ${\left\{\beta_{r}(s)\right\}}_{s,r}$ even if $\text{Cov}\left(Y_{i}|X_{i}\right)$ is misspecified. Specifically, the mean model parameters θ are estimated as the root of the penalized estimating equation

(2) $$\sum_{i=1}^{N}U_{\Lambda }\left(X_{i},Y_{i};\theta_{\Lambda }\right)≔\sum_{i=1}^{N}D_{i}^{T}V_{i}^{-1}\left(Y_{i}-\mu_{i}\right)-\Lambda S\theta_{\Lambda },$$

where $V_{i}\in R^{n_{i}L\times n_{i}L}$ is the working covariance matrix for cluster i (whose true covariance matrix is $V_{i}^{*}$), $D_{i}=\frac{\partial \mu_{i}(\theta )}{\partial \theta },X_{i}={\left[X_{i,1}^{T},\ldots ,X_{i,n_{i}}^{T}\right]}^{T}\in R^{n_{i}L\times p}$, and $\mu_{i}={\left[\mu_{i,1}^{T},\ldots ,\mu_{i,n_{i}}^{T}\right]}^{T}\in R^{n_{i}L}$. The pre-specified penalty matrix, $S\in R^{p\times p}$, is associated with the diagonal matrix of smoothing parameters $\Lambda \in R^{p\times p}$. Although no likelihood is adopted, the estimating equation (2) can be derived from the score equations from, for example, an exponential dispersion family (Liang and Zeger, 1986); we add the penalty term for improved estimation in finite samples. Compared to a working independence matrix $V_{i}=I_{n_{i}L}$, the estimation of θ can be made more efficient and accurate by exploiting correlation, in both functional and longitudinal directions, by choosing the working covariance matrix $V_{i}$ to estimate $V_{i}^{*}$. Although such choices for $V_{i}$ in this fGEE model yield desirable statistical properties for longitudinal FDA, estimation is computationally intensive: estimating θ based on equation (2) requires inversion of the $n_{i}L\times n_{i}L$ covariance matrix $V_{i}$ for each cluster i, at each step in an optimization procedure.

### 2.2 One-step fGEE

To scale fGEE to large datasets, we propose a one-step estimator of the form

(3) $${\hat{\theta }}_{\Lambda_{1}}^{\left(1\right)}={\hat{\theta }}_{\Lambda_{0}}^{\left(0\right)}-{\left[\hat{E}\left(\nabla_{\theta }U_{\Lambda_{1}}\left(X_{i},Y_{i};{\hat{\theta }}_{\Lambda_{0}}^{\left(0\right)}\right)\right)\right]}^{-1}\frac{1}{N}\sum_{i=1}^{N}U_{\Lambda_{1}}\left(X_{i},Y_{i};{\overset{ˆ}{\theta }}_{\Lambda_{0}}^{\left(0\right)}\right),$$

where ${\hat{\theta }}_{\Lambda_{0}}^{\left(0\right)}$ is an initial estimate fit with smoothing parameters $\Lambda_{0}$. We estimate (3) as

(4) $${\hat{\theta }}_{\Lambda_{1}}^{\left(1\right)}={\hat{\theta }}_{\Lambda_{0}}^{\left(0\right)}+{\left[\frac{1}{N}\sum_{i=1}^{N}{\left[D_{i}\left({\hat{\theta }}_{\Lambda_{0}}^{\left(0\right)}\right)\right]}^{T}{\left[{\hat{V}}_{i}\left({\hat{\theta }}_{\Lambda_{0}}^{\left(0\right)}\right)\right]}^{-1}\left[D_{i}\left({\hat{\theta }}_{\Lambda_{0}}^{\left(0\right)}\right)\right]+\Lambda_{1}S\right]}^{-1}\times \frac{1}{N}\sum_{i=1}^{N}\left\{{\left[D_{i}\left({\hat{\theta }}_{\Lambda_{0}}^{\left(0\right)}\right)\right]}^{T}{\left[{\hat{V}}_{i}\left({\hat{\theta }}_{\Lambda_{0}}^{\left(0\right)}\right)\right]}^{-1}\left[Y_{i}-{\hat{\mu }}_{i}\left({\hat{\theta }}_{\Lambda_{0}}^{\left(0\right)}\right)\right]-\Lambda_{1}S{\hat{\theta }}_{\Lambda_{0}}^{\left(0\right)}\right\},$$

where we plug in ${\hat{\theta }}_{\Lambda_{0}}^{\left(0\right)}$ to calculate $D_{i}\left({\hat{\theta }}_{\Lambda_{0}}^{\left(0\right)}\right),{\hat{V}}_{i}\left({\hat{\theta }}_{\Lambda_{0}}^{\left(0\right)}\right)$, and ${\hat{\mu }}_{i}\left({\hat{\theta }}_{\Lambda_{0}}^{\left(0\right)}\right)=g^{-1}\left(X_{i}{\hat{\theta }}_{\Lambda_{0}}^{\left(0\right)}\right)$, with $g^{-1}$ applied component-wise. The updated estimate for the functional coefficient of covariate r is then obtained as ${\hat{\beta }}_{\Lambda_{1},r}^{\left(1\right)}=B{\hat{\theta }}_{\Lambda_{1},r}^{\left(1\right)}$. Any consistent estimator for θ can be used for ${\hat{\theta }}_{\Lambda_{0}}^{\left(0\right)}$; we use (2) with a working independence correlation structure, i.e., a function-on-scalar regression that ignores within-cluster correlation of outcome values across s and j, and uses the same penalty and spline bases as the fGEE. In practice, we estimate ${\hat{\theta }}_{\Lambda_{0}}^{\left(0\right)}$ with the pffr function (Scheipl et al., 2015) from the refund package (see Appendix A.3 for details). We formalize the necessary consistency properties of ${\hat{\theta }}_{\Lambda_{0}}^{\left(0\right)}$ for the population parameter, θ in Section 3.

The one-step can be conceptualized as a de-biasing of, or equivalently a single Newton-Raphson step from, the initial estimate ${\hat{\theta }}_{\Lambda_{0}}^{\left(0\right)}$. It is much faster than the fully-iterated fGEE, because it requires inversion of a working covariance matrix only twice per cluster: 1) ${\left[{\hat{V}}_{i}\left({\hat{\theta }}_{\Lambda_{0}}^{\left(0\right)}\right)\right]}^{-1}$ to estimate ${\hat{\theta }}_{\Lambda_{1}}^{\left(1\right)}$, and 2) ${\left[{\hat{V}}_{i}\left({\hat{\theta }}_{\Lambda_{1}}^{\left(1\right)}\right)\right]}^{-1}$ to estimate $\hat{\text{Var}}\left({\hat{\theta }}_{\Lambda_{1}}^{\left(1\right)}\right)$ (see Section 2.5).

### 2.3 Working Correlations

Although fGEE yields valid inference regardless of the $V_{i}$ structure used (under correct mean model specification), the statistical and computational efficiency of fGEE depends heavily on the $V_{i}$ form adopted. Our one-step estimator is often far faster than the fully-iterated fGEE in Chen et al. (2013) but, if $n_{i}L$ is large, it still may not scale. We therefore focus on parametric forms of $V_{i}$ that can be inverted quickly. We show in simulations below that these forms of $V_{i}$ can still yield the gains in statistical efficiency provided by more flexible $V_{i}$ specifications. Our theory and implementation neveretheless apply to a one-step with general forms of $V_{i}$.

#### Parametric Correlation Structures

For scalability, we focus on adoption of a block working covariance matrix: $V_{i}=\text{bdiag}\left[V_{i}\left(s_{1}\right),\ldots ,V_{i}\left(s_{L}\right)\right]$, where $V_{i}(s)$ models $\text{Cov}\left(Y_{i}(s)|X_{i}\right)\in R^{n_{i}\times n_{i}}.$^1^ However, when $n_{i}$ is large, even calculating $V_{i}(s{)}^{-1}$ at one point s is computationally intensive with standard linear algebra routines. Luckily, exchangeable and AR1 working covariance matrices can be inverted efficiently. For example, rewriting $V_{i}(s)=A_{i}^{1/2}(s)R_{i}(s)A_{i}^{1/2}(s)$, where $A_{i}(s)=\text{diag}\left(v_{i,1}(s),\ldots ,v_{i,n_{i}}(s)\right)$ and $v_{i,j}(s)$ models $\text{Var}\left(Y_{i,j}(s)|X_{i,j}\right)\in R$, the inverse of $V_{i}(s)$ with exchangeable structure has the analytical form (Lipsitz et al., 2017)

$$V_{i}(s{)}^{-1}=\frac{1}{1-\rho \left(s\right)}A_{i}(s{)}^{-1}-\frac{\rho \left(s\right)}{\left(1-\rho \left(s\right)\right)\left[\left(1-\rho \left(s\right)\right)+n_{i}\rho \left(s\right)\right]}\left(A_{i}(s{)}^{-\frac{1}{2}}1_{n_{i}}\right){\left(A_{i}(s{)}^{-\frac{1}{2}}1_{n_{i}}\right)}^{T},$$

where $1_{n_{i}}$ is a vector of ones, and ρ(s)∈[-1,1) is the intra-cluster correlation coefficient.

If $V_{i}(s)$ has the AR1 structure $\text{Cor}\left(Y_{i,j}(s),Y_{i,j^{\prime }}(s)|X_{i,j}\right)=\rho (s{)}^{\left|j-j^{\prime }\right|},V_{i}^{-1}(s)$ can be efficiently computed because, for ρ(s)≥0, its decomposition yields a Toeplitz $R_{i}(s)$. For an estimated ${\hat{r}}_{i}(s)=\left[Y_{i}(s)-{\hat{\mu }}_{i}(s)\right]{\hat{A}}_{i}(s{)}^{-1/2}$, we can quickly calculate ${\hat{V}}_{i}(s{)}^{-1}{\hat{r}}_{i}(s)$ by solving the Toeplitz system $a={\hat{R}}_{i}(s){\hat{r}}_{i}(s)$ with, for example, the generalized Schur algorithm (Ammar and Gragg, 1988). This is done without fully constructing the $n_{i}\times n_{i}$ matrix ${\hat{V}}_{i}(s)$. When data are observed irregularly, one can use the algorithm proposed in Allévius (2018).

#### Correlation parameters

When adopting the block exchangeable or AR1 correlation structures, each $V_{i}(s)$ is a function of a nuisance correlation parameter ρ(s). We estimate these at each point s separately and then optionally smooth over the functional domain to reduce variability. Defining residuals as $e_{i,j}(s)=\frac{Y_{i,j}(s)-\mu_{i,j}(s)}{\sqrt{v\left(\mu_{i,j}(s)\right)}}$, we use the method of moments estimator for the exchangeable structure (Molenberghs et al., 2005): $\overset{ˆ}{\rho }(s)=\frac{1}{N}\sum_{i=1}^{N}\frac{1}{n_{i}\left(n_{i}-1\right)}\sum_{j\leq k}{\overset{ˆ}{e}}_{i,j}(s){\overset{ˆ}{e}}_{i,k}(s)$, and truncate the $\hat{\rho }(s)$ at 1-ϵ or -1+ϵ if they fall outside the (−1, 1) range. For an AR1 structure, we estimate each $\rho_{i}(s)$ with the Yule-Walker equations (Yule, 1927; Walker, 1931) when the longitudinal observations are sampled at regular time intervals. If sampled irregularly, we estimate $\rho_{i}(s)$ with the MLE estimator proposed in Allévius (2018). We then calculate $\hat{\rho }(s)=\frac{1}{N}\sum_{i=1}^{N}{\hat{\rho }}_{i}(s)$ and truncate the $\hat{\rho }(s)$ at 0 or 1-ϵ if they fall outside the [0, 1) range. The $\overset{ˆ}{\rho }(s)$ are calculated twice in our framework: 1) first using ${\hat{\theta }}_{\Lambda_{0}}^{\left(0\right)}$ to calculate ${\hat{V}}_{i}\left({\hat{\theta }}_{\Lambda_{0}}^{\left(0\right)}\right)$ that is plugged into the one-step estimator (3), and 2) second using ${\hat{\theta }}_{\Lambda_{1}}^{\left(1\right)}$ to calculate ${\hat{V}}_{i}\left({\hat{\theta }}_{\Lambda_{1}}^{\left(1\right)}\right)$ that is plugged into the $\hat{\text{Var}}\left({\hat{\beta }}_{\Lambda_{1}}^{\left(1\right)}\right)$ estimator (see expression (6)).

### 2.4 Tuning Λ

To calculate an initial estimate of ${\hat{\theta }}_{\Lambda_{0}}^{\left(0\right)}$, we select the smoothing parameters, denoted as $\Lambda_{0}$, with fast restricted maximum likelihood (Wood, 2011). We found, however, that calculating the one-step estimate with the same $\Lambda_{0}$ values (i.e. $\theta_{\Lambda_{0}}^{\left(1\right)}$) tends to produce inaccurate coefficient estimates. Therefore, we propose to tune the smoothing parameters for the one-step, denoted as $\Lambda_{1}$, based on the cross-validated prediction performance of the one-step estimator. In our simulations, K-fold cluster cross-validation (CV) produced one-step estimates with better estimation accuracy than one-step estimates based on smoothing parameters selected with GCV^2^ or the bootstrap-based procedure proposed in Chen et al. (2013). We use the negative log-likelihood as a CV fit criteria. We propose the following scalable CV for large datasets.

We define the folds, $\left\{{𝒦}_{1},\ldots ,{𝒦}_{K}\right\}$, as a disjoint partition of cluster index sets (i.e. the held-out cluster indices) where, for $K\geq 2,{𝒦}_{k}\subset [N]$ for each $k\in [K],\underset{k}{⋃}{𝒦}_{k}=[N]$, and ${𝒦}_{k_{1}}\cap {𝒦}_{k_{2}}=\emptyset$ for all $k_{1}\neq k_{2}$. To scale CV to large datasets, we exploit four features of the problem structure. First, each fold’s one-step estimate is calculated with pre-computable quantities. For example, rewriting the update as

$${\hat{\theta }}_{\Lambda_{1}}^{\left(1\right)}={\hat{\theta }}_{\Lambda_{0}}^{\left(0\right)}+{\left[\frac{1}{N}\sum_{i=1}^{N}W_{i}\left({\hat{\theta }}_{\Lambda_{0}}^{\left(0\right)}\right)+\Lambda_{1}S\right]}^{-1}\frac{1}{N}\sum_{i=1}^{N}\left\{b_{i}\left({\hat{\theta }}_{\Lambda_{0}}^{\left(0\right)}\right)-\Lambda_{1}S{\hat{\theta }}_{\Lambda_{0}}^{\left(0\right)}\right\},$$

illustrates that we can pre-compute each cluster’s $W_{i}\left({\hat{\theta }}_{\Lambda_{0}}^{\left(0\right)}\right)={\left[D_{i}\left({\hat{\theta }}_{\Lambda_{0}}^{\left(0\right)}\right)\right]}^{T}{\left[{\hat{V}}_{i}\left({\hat{\theta }}_{\Lambda_{0}}^{\left(0\right)}\right)\right]}^{-1}\left[D_{i}\left({\hat{\theta }}_{\Lambda_{0}}^{\left(0\right)}\right)\right]\in R^{p\times p}$, and $b_{i}\left({\hat{\theta }}_{\Lambda_{0}}^{\left(0\right)}\right)={\left[D_{i}\left({\hat{\theta }}_{\Lambda_{0}}^{\left(0\right)}\right)\right]}^{T}{\left[{\hat{V}}_{i}\left({\hat{\theta }}_{\Lambda_{0}}^{\left(0\right)}\right)\right]}^{-1}\left[Y_{i}-{\hat{\mu }}_{i}\left({\hat{\theta }}_{\Lambda_{0}}^{\left(0\right)}\right)\right]\in R^{p}$. Second, we only need to estimate ${\hat{\theta }}_{\Lambda_{0}}^{\left(0\right)}$ once. We can then use that ${\hat{\theta }}_{\Lambda_{0}}^{\left(0\right)}$, calculated on the full sample, as the initial estimate for all folds and $\Lambda_{1}$ values. This is because any consistent initial estimate, ${\hat{\theta }}_{\Lambda_{0}}^{\left(0\right)}$, is sufficient to ensure that the one-step estimator of a given fold is consistent for the population θ. This strategy may be unnecessary for datasets where K fold-specific initial estimates can be calculated quickly. Third, assuming $\frac{1}{N}\sum_{i=1}^{N}W_{i}\left({\hat{\theta }}_{\Lambda_{0}}^{\left(0\right)}\right)+\Lambda_{1}\overset{P}{\to }E\left[\nabla_{\theta }U_{\Lambda_{1}}\left(X_{i},Y_{i};\theta_{\Lambda_{0}}\right)\right]$, we can (heuristically, by Slutsky’s theorem) calculate consistent one-step estimates in fold k as

(5) $${\hat{\theta }}_{\Lambda_{1}}^{k}={\hat{\theta }}_{\Lambda_{0}}^{\left(0\right)}+{\left[\frac{1}{N}\sum_{i=1}^{N}W_{i}\left({\hat{\theta }}_{\Lambda_{0}}^{\left(0\right)}\right)+\Lambda_{1}S\right]}^{-1}\frac{1}{N}\sum_{i\notin {𝒦}_{k}}\left\{{\tilde{n}}_{k}b_{i}\left({\hat{\theta }}_{\Lambda_{0}}^{\left(0\right)}\right)-\Lambda_{1}S{\hat{\theta }}_{\Lambda_{0}}^{\left(0\right)}\right\},$$

where ${\tilde{n}}_{k}=\frac{\sum_{i=1}^{N}n_{i}}{\sum_{i\notin {𝒦}_{k}}n_{i}}$. By using the full sample estimate ${\left[\frac{1}{N}\sum_{i=1}^{N}W_{i}\left({\hat{\theta }}_{\Lambda_{0}}^{\left(0\right)}\right)+\Lambda_{1}S\right]}^{-1}$, we only need to invert this p×p matrix once for each value of $\Lambda_{1}$, instead of inverting a fold-specific p×p matrix for each unique $\left\{k,\Lambda_{1}\right\}$ pair. The strategy of keeping ${\hat{\theta }}_{\Lambda_{0}}^{\left(0\right)}$ and ${\left[\frac{1}{N}\sum_{i=1}^{N}W_{i}\left({\hat{\theta }}_{\Lambda_{0}}^{\left(0\right)}\right)+\Lambda_{1}S\right]}^{-1}$ fixed across folds is motivated by an analogous strategy for cluster bootstrapping of unpenalized one-step GEE (see Remark and Theorem 3.3 in Cheng et al. (2013)). Specifically, Cheng et al. (2013) showed that a cluster bootstrap that fixes these two quantities (at the full-sample estimates) across replicates enjoys the same theoretical guarantees asymptotically as an approach that re-estimates these quantities in each replicate-specific sample. In our simulations, our adaptation of this strategy for cluster CV was often dramatically faster than, and performed nearly identically to, a CV strategy that calculates ${\hat{\theta }}_{\Lambda_{1}}^{k}$ using the fold-specific estimate ${\left[\frac{1}{N-\left|{𝒦}_{k}\right|}\sum_{i\notin {𝒦}_{k}}W_{i}\left({\hat{\theta }}_{\Lambda_{0}}^{\left(0\right)}\right)+\left(\frac{\sum_{i\notin {𝒦}_{k}}n_{i}}{\sum_{i=1}^{N}n_{i}}\right)\Lambda_{1}S\right]}^{-1}$. Fourth, we avoid tuning over a large grid of $\Lambda_{1}$ values by using a sequential CV procedure (see Appendix A.1 for details). We found these strategies performed well with K=10 in our simulations and data application.

### 2.5 Coefficient Estimator Variance

#### 2.5.1 Sandwich Variance Estimator

We estimate the variance of our one-step estimator using the sandwich form

(6) $${\hat{V}}_{\Lambda }\left(\theta \right)=\frac{1}{N}{\left[{\hat{H}}_{\Lambda }\left(\theta \right)\right]}^{-1}{\hat{M}}_{\Lambda }\left(\theta \right){\left[{\hat{H}}_{\Lambda }\left(\theta \right)\right]}^{-1},$$

where ${\hat{M}}_{\Lambda }(\theta )=P_{N}\left[U_{\Lambda }\left(X_{i},Y_{i};\theta \right)U_{\Lambda }{\left(X_{i},Y_{i};\theta \right)}^{T}\right]$, and ${\hat{H}}_{\Lambda }(\theta )=P_{N}\left[D_{i}(\theta {)}^{T}{\hat{V}}_{i}(\theta {)}^{-1}D_{i}(\theta )\right]+\Lambda S$, and $P_{N}\left[f\left(O_{i}\right)\right]=\frac{1}{N}\sum_{i=1}^{N}f\left(O_{i}\right)$ denotes the sample average of a given function f. We estimate $\hat{\text{Var}}\left({\hat{\theta }}_{\Lambda_{1}}^{\left(1\right)}\right)$ via ${\hat{V}}_{\Lambda_{1}}\left({\hat{\theta }}_{\Lambda_{1}}^{\left(1\right)}\right)$, i.e., by plugging in ${\hat{\theta }}_{\Lambda_{1}}^{\left(1\right)},{\hat{V}}_{i}\left({\hat{\theta }}_{\Lambda_{1}}^{\left(1\right)}\right),{\hat{A}}_{i}\left({\hat{\theta }}_{\Lambda_{1}}^{\left(1\right)}\right)$, and $\Lambda_{1}$.

#### 2.5.2 Fast Cluster Bootstrap Variance Estimator

Motivated by theory for cluster bootstrapping in non-functional one-step GEE (Cheng et al., 2013), we propose a fast cluster bootstrap as an alternative method to estimate $\text{Var}\left({\hat{\theta }}_{\Lambda_{1}}^{\left(1\right)}\right)$, or to construct non-parametric bootstrap-based joint CIs. Namely, for bootstrap replicate, t

(7) $${\hat{\theta }}_{\Lambda_{1}}^{t}={\hat{\theta }}_{\Lambda_{1}}^{\left(0\right)}+{\left[\frac{1}{N}\sum_{i=1}^{N}W_{i}\left({\hat{\theta }}_{\Lambda_{0}}^{\left(0\right)}\right)+\Lambda_{1}S\right]}^{-1}\frac{1}{N}\sum_{i\in {ℛ}_{t}}\left\{{\tilde{n}}_{t}b_{i}\left({\hat{\theta }}_{\Lambda_{0}}^{\left(0\right)}\right)-\Lambda_{1}S{\hat{\theta }}_{\Lambda_{0}}^{\left(0\right)}\right\},$$

where ${ℛ}_{t}$ is a set of cluster indices of size N, sampled with replacement, and ${\tilde{n}}_{t}=\frac{\sum_{i=1}^{N}n_{i}}{\sum_{i\in {ℛ}_{t}}n_{i}}$. We estimate ${\text{Var}}_{\text{boot}}\left({\hat{\theta }}_{\Lambda }^{\left(1\right)}\right)$ as the sample covariance matrix of the T bootstrap replicates. Since equation (7) uses the same initial estimate ${\hat{\theta }}_{\Lambda_{1}}^{\left(0\right)}$ and keeps the matrix ${\left[\frac{1}{N}\sum_{i=1}^{N}W_{i}\left({\hat{\theta }}_{\Lambda_{0}}^{\left(0\right)}\right)+\Lambda_{1}S\right]}^{-1}$ fixed for all t, this bootstrapping procedure typically takes less than a second for moderately sized p. We show in simulations that coverage is comparable between CIs constructed with sandwich and fast bootstrap variance estimators (see Appendix Table 20).

### 2.6 Confidence Intervals

For fixed basis matrix $B,\hat{\text{Var}}(\hat{\beta })=\text{blockdiag}\left({\hat{\Sigma }}_{1}^{\left(\beta \right)},\ldots ,{\hat{\Sigma }}_{q}^{\left(\beta \right)}\right)=\left(I_{q}⊗B\right)\hat{\text{Var}}\left({\hat{\theta }}_{\Lambda_{1}}^{\left(1\right)}\right){\left(I_{q}⊗B\right)}^{T}$, where ($I_{q}⊗B$) is a block diagonal matrix, with B in each block. An asymptotically valid (1-α)-level *pointwise* CI is given by ${\hat{\beta }}_{r}(s)\pm z_{1-\alpha /2}{\overset{ˆ}{\sigma }}_{r}^{\left(\beta \right)}(s)$, where ${\overset{ˆ}{\sigma }}_{r}^{\left(\beta \right)}(s)=\sqrt{{\hat{\Sigma }}_{r}^{\left(\beta \right)}(s)}$, and ${\hat{\Sigma }}_{r}^{\left(\beta \right)}(s)\in R$ is diagonal entry s of ${\hat{\Sigma }}_{r}^{\left(\beta \right)}$.

We adapt parametric and non-parametric bootstrap based strategies described in Degras (2017) to construct (1-α)-level *joint* CIs. Briefly, we calculate these at point s as ${\hat{\beta }}_{r}(s)\pm {\tilde{q}}_{1-\alpha }^{\left(r\right)}{\overset{ˆ}{\sigma }}_{r}^{\left(\beta \right)}(s)$, where we estimate ${\tilde{q}}_{1-\alpha }^{\left(r\right)}$ as the 1-α empirical quantile of statistics calculated on a bootstrap sample, ${\left\{{\tilde{\theta }}_{r}^{t}\right\}}_{t=1}^{T}$, drawn from the sampling distribution of ${\hat{\theta }}_{\Lambda_{1}}^{\left(1\right)}$. For the parametric approach, we sample ${\tilde{\theta }}_{r}^{t}\sim N_{m}\left(0,{\hat{\Sigma }}_{r}^{\left(\theta \right)}\right)$, with ${\hat{\Sigma }}_{r}^{\left(\theta \right)}$ denoting the m×m submatrix of $\hat{\text{Var}}\left({\hat{\theta }}_{\Lambda_{1}}^{\left(1\right)}\right)$ associated with covariate r. For the non-parametric approach, we calculate ${\tilde{\theta }}_{r}^{t}$ with our fast cluster bootstrap. We then set $m_{r}^{t}=\text{max}\left(\left|{\tilde{\theta }}_{r}^{t}\right|⊘\text{diag}\left({\hat{\Sigma }}_{r}^{\left(\theta \right)}\right)\right)$, where ⊘ denotes element-wise division, and estimate ${\tilde{q}}_{1-\alpha }^{\left(r\right)}$ as the 1-α empirical quantile of ${\left\{m_{r}^{t}\right\}}_{t=1}^{T}$. Estimating ${\tilde{q}}_{1-\alpha }^{\left(r\right)}$ based on draws of ${\tilde{\theta }}_{r}^{t}$, instead of draws of ${\tilde{\beta }}_{r}^{t}$, is much faster as usually $\text{dim}\left(\theta_{r}\right)\ll \text{dim}\left(\beta_{r}\right)$. We apply the parametric approach in results shown in the main text, but provide simulation results in Appendix C.4 that show the non-parametric strategy achieves similar joint coverage.

## 3 Theory

For a fixed sequence $\Lambda_{N}=\text{diag}\left(\lambda_{N,1},\ldots ,\lambda_{N,p}\right)$ with $\frac{1}{N}{\text{max}}_{1\leq j\leq p}\lambda_{N,p}\to 0$, let $\theta_{N}$ be the solution to the population estimating equation $E\left\{U_{N}\left(X_{i},Y_{i};\theta \right)\right\}=0$, where $U_{N}\left(X_{i},Y_{i};\theta \right)=D_{i}^{T}(\theta )V_{i}^{-1}\left(Y_{i}-\mu_{i}(\theta )\right)-\frac{1}{N}\Lambda_{N}S\theta$ has components denoted $U_{N,j}\left(X_{i},Y_{i};\theta \right)$ for j∈[p], and let $\beta_{N}=\left(I_{q}⊗B\right)\theta_{N}$. We define $H_{N}(\theta )=E\left[D_{i}^{T}(\theta )V_{i}^{-1}D_{i}(\theta )+\frac{1}{N}\Lambda_{N}S\right]$, and $M_{N}(\theta )=E\left[U_{N}\left(X_{i},Y_{i};\theta \right)U_{N}{\left(X_{i},Y_{i};\theta \right)}^{T}\right]$. Note that implicit to these definitions, and to the ensuing theory, is that we treat the working covariance matrices $V_{i}$ as fixed or computed with the “true” limiting correlation parameters ρ(s) throughout. In practice, we can replace these with their estimated counterparts ${\hat{V}}_{i}$ under the assumption that $\hat{\rho }(s)=\rho (s)+o_{P}\left(n^{-1/2}\right)$ for all s, for some limiting parameters ρ(s)—see Corollary 1 of Chen et al. (2013).

**Theorem 3.1**. *Suppose that the one-step estimators*, ${\hat{\theta }}_{\Lambda_{N}}^{\left(1\right)}$ *and* ${\hat{\beta }}_{\Lambda_{N}}^{\left(1\right)}$, *are constructed using initial estimate*, ${\hat{\theta }}_{N}^{\left(0\right)}$*, and that the following conditions hold:*

- The inverse link function $g^{-1}$ is three times continuously differentiable.
- *The covariates and outcomes have bounded support, i.e*. ∃M>0 *such that* $P\left[\left\|Y_{i}(s)\right\|\leq \right.M]=1$ *for all* s∈𝒮, *and* $P\left[\left|X_{i,j,r}\right|<M\right]=1$, *for all* $j\in \left[n_{i}\right]$ *and* r∈[q].
- $\exists s,t>0:\lambda_{\text{min}}\left(M_{N}\left(\theta_{N}\right)\right)\geq s,E\left(\left|U_{N,j}\left(X_{i},Y_{i};\theta \right)U_{N,k}\left(X_{i},Y_{i};\theta \right)U_{N,l}\left(X_{i},Y_{i};\theta \right)\right|\right)\leq t,\forall n\in N,\forall j,k,l$.
- $H_{N}\left(\theta_{N}\right)=is\;\text{invertible},P\left[P_{N}\left({\left.\nabla_{\theta }U_{N}\left(X_{i},Y_{i};\theta \right)\right|}_{\theta ={\hat{\theta }}_{N}^{\left(0\right)}}\right)\right.\;\text{non-singular}]=1$, *and* ${\left(P_{N}\left[{\left.\nabla_{\theta }U_{N}\left(X_{i},Y_{i};\theta \right)\right|}_{\theta ={\hat{\theta }}_{N}^{\left(0\right)}}\right]\right)}^{-1}=O_{P}(1)$.
- $M_{N}\left(\theta_{N}\right)=O(1)$, $H_{N}\left(\theta_{N}\right)=O(1)$ *and* ${\left\{H_{N}\left(\theta_{N}\right)\right\}}^{-1}=O(1)$.
- $\sqrt{N}{\left\{M_{N}\left(\theta_{N}\right)\right\}}^{-1/2}H_{N}\left(\theta_{N}\right)\left({\hat{\theta }}_{N}^{\left(0\right)}-\theta_{N}\right)=O_{P}(1)$.

*Then the one-step estimator satisfies $\sqrt{N}{\left\{V_{N}\right\}}^{-1/2}\left({\hat{\beta }}_{\Lambda_{N}}^{\left(1\right)}-\beta_{N}\right)\overset{d}{\to }𝒩\left(0,I_{p}\right)$*, *where* $V_{N}=\left(I_{q}⊗\right.\text{B}){\left\{H_{N}\left(\theta_{N}\right)\right\}}^{-1}M_{N}\left(\theta_{N}\right){\left\{H_{N}\left(\theta_{N}\right)\right\}}^{-1}{\left(I_{q}⊗B\right)}^{T}$.

Condition (i) is a mild smoothness condition that holds for all standard link functions (e.g. logit, log). Condition (ii) is also standard—we expect it holds across essentially all biomedical settings. Note that it could be replaced by weaker moments conditions on the estimating equation, and its derivatives. Condition (iii) is a sufficient condition for the estimating equation to be asymptotically normal and implies that $M_{N}\left(\theta_{N}\right)$ is invertible for all N∈N. Condition (iv) also states that the $H_{N}\left(\theta_{N}\right)$, and its sample analogue, are invertible for all N∈N. Condition (v) should hold when the limiting (unpenalized) estimating equation results in full rank limiting $M_{N}$ and $H_{N}$. This should hold when the design matrices, $X_{i}$, are full rank. Finally condition (vi) is a statement about the rate of convergence of the initial estimator. In practice, when the ${\hat{\theta }}_{N}^{\left(0\right)}$ is estimated using a penalized unweighted estimating equation, this implies some conditions on the rates of convergence of the smoothing parameter values, $\Lambda_{0,N}$ and $\Lambda_{N}$. We provide an expanded discussion of this in Appendix B, where we also develop a more general result for adaptive one-step M-estimation that may be of independent interest.

**Remark 3.2**. *Our result shows that the one-step is asymptotically equivalent to the fully-iterated fGEE. Moreover, our result extends to non-linear link functions, while existing fGEE theory is restricted to the linear case* (Chen et al., 2013). *In the linear case, the one-step shares the same properties as those characterized in* Chen et al. (2013), *such as the convergence rates in small knot and large knot regimes in terms of the smoothing parameter*, Λ. *We provide a lengthier discussion of the convergence rates of the coefficient estimates in terms of the smoothing parameters in* Appendix B.

## 4 Simulations

We conducted simulations to assess 95% CI coverage, coefficient estimate accuracy, and algorithm timing. We report results from T=300 simulation replicates. We fit function-on-scalar regressions using penalized B-splines with 10 knots per functional coefficient. For both simulations, we set s∈𝒮⊂[0,1], |𝒮|=L=100,N∈{50,100}, and $n_{i}\in \{5,25,100\}$. Denoting ${\hat{\beta }}_{r}^{t}(s)$ as functional coefficient r for simulation replicate t as point s, we report estimation accuracy as $\text{RMSE}=\frac{1}{T}\sum_{t=1}^{T}{\left[\frac{1}{\left(q+1)|𝒮\right|}\sum_{r=0}^{q}\sum_{s\in 𝒮}{\left(\beta_{r}(s)-{\hat{\beta }}_{r}^{t}(s)\right)}^{2}\right]}^{1/2}$. Denoting ${\text{pCI}}_{r}^{t}(s)$ as the pointwise CI for replicate t for functional coefficient r at point s, we report the average empirical pointwise coverage as: $\frac{1}{T(q+1)|𝒮|}\sum_{t=1}^{T}\sum_{r=0}^{q}\sum_{s\in 𝒮}1\left(\beta_{r}(s)\in {\text{pCI}}_{r}^{t}(s)\right)$. Denoting the joint CI as ${\text{jCI}}_{r}^{t}(s)$, we report empirical joint coverage as: $\frac{1}{T(q+1)}\sum_{t=1}^{T}\sum_{r=0}^{q}1\left(\beta_{r}(s)\in {\text{jCI}}_{r}^{t}(s)\forall s\in 𝒮\right)$. In simulation 1, we generated continuous data to allow comparison with existing methods and a penalized Generalized Least Squares (GLS), which is similar to the fully-iterated fGEE. We show that the one-step with the pointwise working correlation structure, that we adopted for scalability, yields gains in statistical efficiency, and performs as well or better (in finite samples) than a method that adopts the true correlation structure in both longitudinal and functional directions. In simulation 2, we verify the one-step’s performance in a binary outcome setting. We include additional simulations in Appendix Section C.1.

### 4.1 Simulation 1: Gaussian Outcome with Exchangeable Correlation

We first tested one-step performance in a setting where the outcome was simulated to be correlated in both longitudinal and functional directions (i.e. $\text{Cov}\left(Y_{i,j}\left(s_{1}\right),Y_{i,j^{\prime }}\left(s_{2}\right)|X_{i}\right)\neq 0$ for $s_{1},s_{2}\in 𝒮$ and, $j^{\prime }\in \left[n_{i}\right]$). Thus this experiment also tests how the one-step performs when the pointwise longitudinal correlation structure we adopt is misspecified (i.e. it ignores the underlying correlation in the functional direction). Specifically, we simulated the outcome with an exchangeable correlation structure, allowing for comparison with the marginal decomposition (“Marginal”) approach proposed in Li et al. (2022), which models both within- and between-functional observation correlation. To provide a fair comparison with the Marginal approach, we based these synthetic data experiments on their marginal decomposition simulation scheme and code. We simulated data with the model

(8) $$Y_{i,j}(s)=\beta_{0}(s)+X_{1,i}\beta_{1}(s)+X_{2,i,j}\beta_{2}(s)+W_{i,j}(s)+\epsilon_{i,j}(s)$$

where $\beta_{0}(s)=3+\text{sin}(\pi s)+\sqrt{2}\text{cos}(3\pi s)$, $\beta_{1}(s)=3+\text{cos}(2\pi s)+\sqrt{2}\text{cos}(3\pi s)$, and $\beta_{2}(s)=\frac{1}{60}\left[\phi \left(\frac{s-0.2}{0.1^{2}}\right)+\phi \left(\frac{s-0.1}{0.07^{2}}\right)\right]-\frac{1}{200}\phi \left(\frac{s-0.35}{0.1^{2}}\right)-\frac{1}{250}\phi \left(\frac{s-0.65}{0.06^{2}}\right)$. Based on simulations in Li et al. (2022), we drew $X_{1,i}\sim N(0,1)$, and $X_{2,i,j}=j+e_{i,j}$, where $e_{i,j}\sim N\left(\alpha e_{i,j-1},1\right)$, with $e_{i,0}=0,\alpha =0.7$. We set parameters as in Li et al. (2022): $W_{i,j}(s)=\sum_{k=1}^{2}\left(\xi_{i,k}+\zeta_{i,j,k}\right)\psi_{k}(s)$ where the orthonormal functions $\psi_{1}(s)=1\forall s\in 𝒮$ and $\psi_{2}(s)=\sqrt{2}\text{sin}(2\pi s)$, $\xi_{i,1}\overset{\text{iid}}{\sim }N(0,3)$, $\xi_{i,2}\overset{\text{iid}}{\sim }N(0,2)$, $\zeta_{i,j,1}\overset{\text{iid}}{\sim }N(0,1.5)$ and $\xi_{i,j,2}\overset{\text{iid}}{\sim }N(0,1).\epsilon_{i,j}(s)\overset{\text{iid}}{\sim }N(0,1.5)$. We show results from simulations with different parameters in Appendix C.2.

In addition to the Marginal approach, we compared the one-step to three benchmarks: 1) a penalized GLS with an independence working correlation structure (GLS-Ind), 2) a GLS with an exchangeable correlation structure (GLS-Ex), and 3) the initial function-on-scalar regression (FoSR), ${\hat{\beta }}_{\Lambda_{0}}^{\left(0\right)}$. We constructed CIs with a sandwich variance estimator for all methods, using the corresponding independence or exchangeable $V_{i}$ forms. Benchmark 1) shows how our implementation and tuning scheme performs without exploiting intra-cluster correlation, benchmark 2) shows performance of an estimator similar to a fully-iterated version of the one-step fGEE (using the same exchangeable correlation structure), and benchmark 3) shows the performance of a FoSR that ignores intra-cluster correlation. CIs for the FoSR fit should, however, achieve nominal coverage in this correlated setting, given that we use a sandwich variance estimator. See Appendix Sections A.2 and A.3 for further details on benchmarks 1–3. When analyzing continuous data in practice, the closed-form penalized GLS (e.g. GLS-Ex) is fast to calculate and is thus preferable to the one-step. However, we assess one-step performance in the continuous data setting because the availability of a closed-form estimator allows us to test how our *one-step* fGEE performs relative to a method similar to a *fully-iterated* fGEE.

Table 1 shows that the one-step coefficient accuracy is almost identical to the GLS-Ex, suggesting the one-step performs comparably to a fully-iterated fGEE. Moreover, the one-step performs as well or better than the Marginal estimator in Li et al. (2022). Thus, in these simulations, modeling $\text{Cov}\left(Y_{i,j}(s),Y_{i,j^{\prime }}(s)|X_{i}\right)$ at each point s across values of $j,j^{\prime }\in \left[n_{i}\right]$, is enough to capture efficiency gains, even though the data were simulated such that $\text{Cov}\left(Y_{i,j}\left(s_{1}\right),Y_{i,j^{\prime }}\left(s_{2}\right)|\right.\left.X_{i}\right)\neq 0$, for $s_{1}\neq s_{2}$. Table 2 shows that the pointwise coverage of the one-step is at roughly the nominal levels. In contrast, the Marginal grows highly anti-conservative for large $n_{i}$, a feature acknowledged in Li et al. (2022). GLS-Ind tends to be slightly conservative, while FoSR is slightly anti-conservative at smaller sample sizes. Table 3 shows that the joint CIs^3^ are somewhat conservative for every method, although less so for FoSR. Together these simulations provide one example of how the correlation structure adopted here for computational reasons can still yields gains in statistical efficiency, even when compared to methods that model correlation in both longitudinal and functional directions.

These simulations also illustrate the scalability of the one-step. Table 4 shows that the one-step scales well with both N and $n_{i}$. In contrast, the Marginal approach is too memory-intensive to fit larger datasets and fit-times scale super-linearly as a function of N and $n_{i}$. Appendix A.1.3 has tables comparing performance of the one-step fit with a $\Lambda_{1}$ tuned using the “fast K-fold” and “K-fold” CV strategies. The fast K-fold CV approach yields similar performance, often in a fraction of the time. Tables in Appendix C.3 show that one-step 95% CIs achieve similar coverage, and take similar time to calculate, when constructed with sandwich or fast cluster bootstrap $\hat{\text{Var}}\left({\hat{\beta }}_{\Lambda_{1}}^{\left(1\right)}\right)$ estimators. Finally, we show in Appendix C.4 that Joint CIs constructed with parametric and fast non-parametric bootstrap strategies achieve similar coverage. Taken together, our strategies for working correlation matrix inversion, smoothing parameter tuning, bootstrapping, and joint CI construction yield fast and accurate results.

### 4.2 Simulation 2: Binary Outcome with AR1 Correlation

We simulated correlated binary data with the SimCorMultRes package (Touloumis, 2016) with functional observations observed on an evenly spaced grid. The mean model was

(9) $$\text{logit}\left(E\left(Y_{i,j}(s)|X_{i,j}\right)\right)=\beta_{0}(s)+X_{1,i}\beta_{1}(s)+X_{2,i,j}\beta_{2}(s)$$

where $\beta_{0}(s)=1+\frac{1}{3}\text{sin}(\pi s)+\frac{\sqrt{2}}{3}\text{cos}(3\pi s),\beta_{1}(s)=1+\frac{1}{3}\text{cos}(2\pi s)+\frac{\sqrt{2}}{3}\text{cos}(3\pi s),\beta_{2}(s)=\frac{5}{3}\phi \left(\frac{s-0.35}{0.1}\right)-\frac{5}{3}\phi \left(\frac{s-0.65}{0.2}\right)$, and ϕ(⋅) denotes the standard normal density function. The covariates were drawn as described in Section 4.1. The $n_{i}L\times n_{i}L$ covariance matrix was set as $\text{Cov}\left(Y_{i}|X_{i}\right)=\text{blockdiag}\left(\Sigma_{i}\left(s_{1}\right),\ldots ,\Sigma_{i}\left(s_{L}\right)\right)$, where $\text{Cov}\left(Y_{i}(s)|X_{i}\right)=\Sigma_{i}(s)\in R^{n_{i}\times n_{i}}$. We used the AR1 structure $\text{Cor}\left(Y_{i,j}(s),Y_{i,j^{\prime }}|X_{i,j}\right)=\rho^{\left|j-j^{\prime }\right|}$ for ρ(s)=ρ∈{0.25,0.5,0.75}.

Table 5 shows that the one-step improves coefficient estimation performance relative to the initial FoSR fit, ${\hat{\beta }}_{\Lambda_{0}}^{\left(0\right)}$, particularly when $n_{i}$ and/or ρ(s) is large. Tables 6 and 7 show our approach improves CI coverage relative to FoSR. The one-step coverage hovers around 0.9 (pointwise) and 0.98 (joint). In contrast, the FoSR CI coverage is poor, particularly when both ρ and $n_{i}$ are large. This is unexpected as we use a sandwich variance estimator to construct FoSR CIs (calculated with an independence working correlation), so we expected that they would achieve nominal coverage. Thus, while fitting a FoSR with working independence and using a sandwich estimator for inference may seem like a fast, viable alternative to fGEE (with a working correlation other than independence), it can yield CIs with poor coverage. Finally, the fit times, shown in Table 8, demonstrate the scalability of the one-step estimator.

## 5 Application

We apply our framework to calcium imaging data to illustrate the benefits of longitudinal FDA in analyzing neural recordings. To motivate our approach, we first describe common analysis strategies. There is a rich methodological literature on analyzing neuronal firing data to, for example, identify spike times (Jewell and Witten, 2018), denoise data (Pnevmatikakis et al., 2016), identify network connections (Wang et al., 2025), and model interactions between neurons with dynamical systems (Glaser et al., 2020). These analysis approaches have different goals and we do not review them here due to space constraints. Instead we focus on what we have observed are common strategies among experimentalists for hypothesis testing of covariate-neural activity associations, as they have similar goals to our proposed method. These approaches seem to vary largely in how 1) the target neural population is defined, 2) the longitudinal structure is accounted for, and 3) the trial-level neural time-series are modeled.

**Figure 1:**
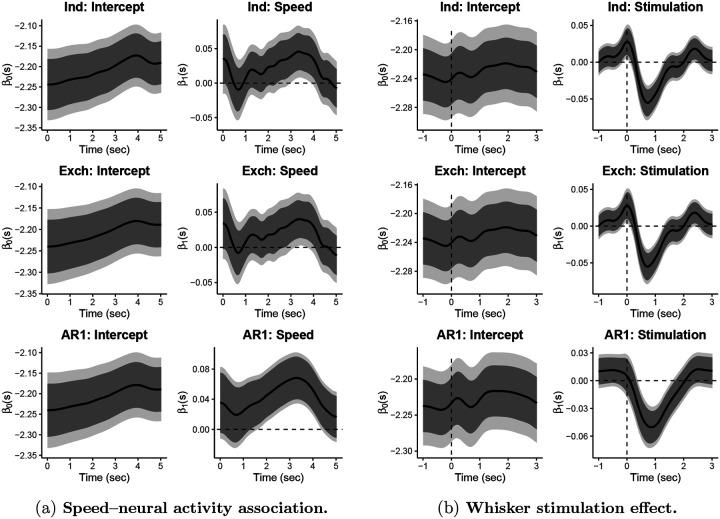
Functional coefficient estimates for Independent (Ind), Exchangeable (Exch), and Auto-regressive-1 (AR1) working correlation structures.

Since calcium imaging and electrophysiology record the activity of many neurons, and recordings are collected in several animals, analyses differ in how the target population is defined and the nesting of neurons within animal is modeled. For example, the *neural pseudo-population* strategy, as we refer to it, fits a single model to a dataset that pools neurons across animals (e.g. see Figures 1, 3, and 3 of Willmore et al. (2023); Zhang et al. (2023); Roesch et al. (2009), respectively). This conceptualizes neurons, both within and across animals, as exchangeable given covariates and model parameters. The *animal-specific neural population* strategy, as we refer to it, summarizes the collection of neurons separately in each animal, and then summarizes the animal-specific statistics with a secondary pooled test statistic (e.g. see Figs 2H in (Legaria et al., 2022), Fig 1G, 1I in Inácio et al. (2025)). The animal-level summary is usually a model fit to, or an average of, the activity of all neurons recorded from that animal (e.g. see Figs 2G in (Legaria et al., 2022)). This ignores uncertainty in the animal-level statistics when estimating a pooled test statistic. A third approach estimates a test statistic on data from each neuron separately and then fits a model to those statistics (e.g. see Figures 1K, 2E-H of Inácio et al. (2025)). The pooled test ignores uncertainty in the neuron-level statistics, and models the neuron-level statistics estimated on data from neurons in the same animal as independent.

Analysis strategies differ in how the longitudinal structure of experiments are modeled. One strategy is to treat the neural responses of cluster i — however defined — as exchangeable across trials given model parameters (e.g. see Figure 3E of Jeong et al. (2022) for an example from photometry). A second strategy averages the response across trials and analyzes those trial-averaged measures (e.g. see Figure 2 of Coddington et al. (2023)). This discards longitudinal information. A third strategy accounts for the longitudinal structure with random effects Loewinger et al. (2025), yielding conditional estimates in binary outcome settings.

Analyses vary in how the densely-sampled neural time-series of each trial is conceptualized. Arguably, the most common strategy analyzes univariate summaries of the time-series (e.g. a trial firing rate for each neuron i and trial $j:{\overset{‾}{Y}}_{i,j}=\frac{1}{\left|𝒮\right|}\sum_{s\in 𝒮}Y_{i,j}(s)$) pooled across animals and/or trials (e.g. see Figures 1, 3, and 3 of Willmore et al. (2023); Zhang et al. (2023); Roesch et al. (2009), respectively). This strategy can obscure behavior–brain associations and substantially change scientific conclusions because it discards timing information about how covariate-outcome relationships evolve across trial timepoints (Loewinger et al., 2025). A second strategy is to retain the time-series structure, but model covariate-neural activity associations as constant across trial timepoints for each neuron. For example, Inácio et al. (2025) has the goal of identifying neurons associated with a particular behavior over time (e.g. see Figures 1–2). To that effect, they fit a Pearson correlation between behavior and neural activity in each cell separately. This is comparable to the linear regression $E\left[Y_{i,j}(s)|x_{i,j}(s)\right]=\gamma_{0}^{\left(i\right)}+\gamma_{1}^{\left(i\right)}x_{i,j}(s)$ (e.g. see figure 1K in Inácio et al. (2025)). This models the covariate–outcome relationship as constant across trial timepoints. A third strategy proposed in (Loewinger et al., 2025) addresses this by modeling each trial as a functional outcome. They demonstrate this strategy on fiber photometry data, a recording technique that yields a single neural signal per animal. It would be desirable to apply longitudinal FDA strategies to other recording techniques, such as calcium imaging or electrophysiology data. This has not been done, to our knowledge, and we believe this is primarily due to the fact that those modalities record potentially tens of thousands of signals per animal, and existing approaches may not scale.

To address these limitations, we apply our fGEE on a dataset that pools neurons across animals (akin to the “neural pseudo-population” strategy). This accounts for the 1) longitudinal and 2) functional nature of the response in each cluster (i.e.neuron), and 3) does not discard uncertainty in the animal-specific estimates in providing an overall *neural pseudo population-level* estimate. This implicitly assumes a covariance structure where correlation between neurons within-animal is negligible. In some cases, it may be preferable to apply a fGEE to the neurons in each animal separately (i.e. the *animal-specific neural population* strategy). This would allow for functional coefficients to differ across animals, but necessitates an approach to construct a pooled estimate of animal-level fits that propagates uncertainty.

### 5.1 Application Background

We apply our method on data from a recent *Nature* paper studying the role of pyramidal neurons in the primary somatosensory cortex (S1) in behavior and sensory input (Inácio et al., 2025). This study recorded neuronal activity in five mice, from 155 – 262 (mean± SEM: 184.4 ± 22.61) neurons per animal. Recording was done in head-fixed animals, running on a ball that tracked their movement speed. The authors were interested in identifying S1 neurons active during spontaneous movements. They tested whether each S1 neuron was associated with running speed, whisker movement, and whisker sensory input.

### 5.2 Identifying neural activity encoding speed information

The correlation analysis used in the original paper could only test the neural activity–speed association on average within trials. In contrast, an FDA approach can test how this association evolves across trial timepoints. To demonstrate that, we randomly sampled N=500 neurons (clusters) from the five animals, and identified five second intervals when animals spontaneously began to run. The start of this running burst was considered a “trial” (experimental replicate) initiation as typically analyzed in neuroscience. Thus the functional outcome for neuron i, on trial j was a binary timeseries vector across five seconds of neural activity measured at 30Hz (starting at the beginning of the running bout to 5 sec after running initiation). This results in a functional outcome measured at an evenly spaced grid of |𝒮|=150 points. Speed, $X_{i,j}(s)\in R$ was defined as a functional covariate and took the same value for all neurons recorded from the same animal. The cluster size was $n_{i}=29$ for all neurons i∈[N]. We fit the model

$$\text{logit}\left(E\left[Y_{i,j,l}(s)|X_{i,j}(s)\right]\right)=\beta_{0}(s)+X_{i,j}(s)\beta_{1}(s),$$

with three possible working correlation structures: independence, exchangeable and AR1. For working independence, we applied the sandwich estimator to the initial FoSR fit, ${\hat{\beta }}_{\Lambda_{0}}^{\left(0\right)}$.

The independence and exchangeable correlations structures yielded similar results and showed a significant effect only briefly around 3–4 seconds (see Figure 1a). The estimated exchangeable correlation parameter $\hat{\rho }(s)\approx 0$ for all s, and thus the exchangeable and working independence fits were similar. In contrast, the AR1 model showed a much wider time-interval during which effects were significant. This model also had larger $\hat{\rho }(s))$ estimates. The AR1 structure is a common correlation structure to adopt in timeseries analysis of neuroscience, and seems more appropriate given that successive trials are recorded close together in time.

The most salient finding was that, even with the AR1 model, the speed–neural activity association does not become significant until about one second after the animals begin to run and that association becomes non-significant fairly quickly. The timing of the association suggests that these neurons are not driving the movement of the animal. This shows how the timing sensitivity of fGEE can help identify the type of cognition or behavior a brain region encodes, which is much harder to do with analyses of trial summary measures.

### 5.3 Whisker Stimulation

The authors of the original paper were also interested in how neural activity in S1 neurons changed as a result of whisker stimulation. The summary analyses they carried out only allowed estimation of the extent to which whisker stimulation changed neural activity on average across within-trial timepoints. To characterize the “temporal dynamics” of the neural response to this manipulation, and demonstrate the scalability of the one-step, we applied our method to activity from N=500 randomly selected neurons (clusters), each with $n_{i}=300$ observations of the functional outcome: 4 sec of neural activity, measured 1 sec before whisker stimulation to 3 sec after (L=120). We fit the following model

$$\text{logit}\left(E\left[Y_{i,j,l}(s)|X_{i,j}\right]\right)=\beta_{0}\left(s\right)+X_{i,j}\beta_{1}\left(s\right),$$

where $X_{i,j}\in \{0,1\}$ is an indicator that neuron i was recorded from an animal that was stimulated on trial j. We fit the model with the same working correlation structures as above.

The one-step estimator, fit to 150,000 functional observations, took ~13.38 min to fit on a MacBook Pro with an Apple M1 Max chip with 64GB of RAM, without parallelization, and maintained a reasonable memory footprint throughout. The coefficient associated with stimulation, ${\hat{\beta }}_{1}(s)$, shows the estimated mean difference in neural activity between stimulated and non-stimulated trials. It appears stimulation leads to a rapid reduction in activity that lasts about two sec (see Figure 1b). The estimated exchangeable correlation parameter $\hat{\rho }(s)=0$ for all s, and thus the exchangeable and working independence fits are identical. This analysis illustrates how the one-step makes it possible to identify a clear temporal profile for the effect of interest, and is scalable enough to allow estimation over a large sample of neurons.

In Appendix D, we conduct an additional analysis on this same dataset to examine the association between whisker activity and neural activity. The results reveal that the association between whisker activity and neural activity is significant throughout the trial.

## 6 Discussion

The proposed one-step fGEE can be applied and extended in a range of related settings. For example, only minor adjustments are needed to extend our code to implement an fGEE using a qausi-likelihood based on other distributions (e.g. gamma, beta). In addition to the scalable working covariance forms focused on here, our implementation can also be applied with working covariances, $V_{i}$, that are estimated more flexibly (e.g. using FPCA). Finally, it should be straightforward to extend our method and code to multivariate functional domains, as well as longitudinal scalar-on-function and function-on-function regressions; we believe parameters from these methods can be estimated through estimating equations with similar forms to the fGEE equations used here. We hope our theoretical guarantees and efficient implementation encourage analysts to apply FDA methods in neuroscience and other settings.

## 7 Software and Reproducibility

Our application and simulation code is in the Github repo: https://github.com/gloewing/fgee_onestep. The development version of the fastFGEE package in R can be found in the Github repo: https://github.com/gloewing/fastFGEE.

## Supplementary Material

1

## Figures and Tables

**Table 1:** Functional Coefficient Estimation Performance (RMSE) of each method relative to the FoSR fit ($\text{RMSE}/{\text{RMSE}}_{\text{FoSR}}$). Outcomes are simulated as Gaussian with exchangeable correlation. Cells contain the average of 300 replicates ± SEM (SEM= 0.00 indicates a value < 0.01). We indicate out-of-memory (30Gb) with symbol —.

N	$n_{i}$	One-step	GLS-Ex	GLS-Ind	Marginal
25	5	0.95 ± 0.01	0.95 ± 0.01	0.99 ± 0.00	0.96 ± 0.01
	25	0.97 ± 0.01	0.97 ± 0.01	1.00 ± 0.00	1.01 ± 0.01
	100	1.00 ± 0.00	1.00 ± 0.00	1.01 ± 0.00	1.02 ± 0.01
50	5	0.94 ± 0.01	0.94 ± 0.01	0.99 ± 0.00	0.95 ± 0.01
	25	0.97 ± 0.00	0.97 ± 0.00	1.00 ± 0.00	0.99 ± 0.01
	100	0.99 ± 0.00	0.99 ± 0.00	1.00 ± 0.00	1.04 ± 0.01
100	5	0.94 ± 0.01	0.94 ± 0.01	0.99 ± 0.00	0.93 ± 0.01
	25	0.97 ± 0.00	0.97 ± 0.00	0.99 ± 0.00	0.98 ± 0.01
	100	0.99 ± 0.00	0.99 ± 0.00	1.00 ± 0.00	—

**Table 2:** Pointwise 95% CI coverage. Cells contain the average of 300 replicates ± SEM (SEM= 0.00 indicates a value < 0.01). We indicate out-of-memory (30Gb) with symbol —.

N	$n_{i}$	One-step	GLS-Ex	GLS-Ind	Marginal	FoSR
25	5	0.92 ± 0.00	0.96 ± 0.00	0.96 ± 0.00	0.94 ± 0.00	0.90 ± 0.00
	25	0.92 ± 0.00	0.95 ± 0.00	0.96 ± 0.00	0.94 ± 0.00	0.92 ± 0.00
	100	0.94 ± 0.00	0.97 ± 0.00	0.96 ± 0.00	0.71 ± 0.00	0.93 ± 0.00
50	5	0.95 ± 0.00	0.97 ± 0.00	0.97 ± 0.00	0.94 ± 0.00	0.91 ± 0.00
	25	0.95 ± 0.00	0.97 ± 0.00	0.97 ± 0.00	0.95 ± 0.00	0.94 ± 0.00
	100	0.94 ± 0.00	0.96 ± 0.00	0.97 ± 0.00	0.70 ± 0.00	0.93 ± 0.00
100	5	0.96 ± 0.00	0.98 ± 0.00	0.97 ± 0.00	0.95 ± 0.00	0.92 ± 0.00
	25	0.96 ± 0.00	0.98 ± 0.00	0.98 ± 0.00	0.95 ± 0.00	0.94 ± 0.00
	100	0.96 ± 0.00	0.97 ± 0.00	0.98 ± 0.00	—	0.95 ± 0.00

**Table 3:** Joint 95% CI coverage for Gaussian data simulated with exchangeable correlation. Each cell contains the average of 300 replicates ± SEM (SEM= 0.00 indicates a value < 0.01).

N	$n_{i}$	One-step	GLS-Ex	GLS-Ind	FoSR
25	5	0.97 ± 0.00	0.99 ± 0.00	0.98 ± 0.00	0.95 ± 0.00
	25	0.97 ± 0.00	0.98 ± 0.00	0.98 ± 0.00	0.97 ± 0.00
	100	0.98 ± 0.00	0.99 ± 0.00	0.99 ± 0.00	0.97 ± 0.00
50	5	0.98 ± 0.00	0.99 ± 0.00	0.99 ± 0.00	0.94 ± 0.00
	25	0.99 ± 0.00	0.99 ± 0.00	0.99 ± 0.00	0.98 ± 0.00
	100	0.98 ± 0.00	0.99 ± 0.00	0.99 ± 0.00	0.97 ± 0.00
100	5	0.99 ± 0.00	0.99 ± 0.00	0.99 ± 0.00	0.96 ± 0.00
	25	0.99 ± 0.00	0.99 ± 0.00	0.99 ± 0.00	0.98 ± 0.00
	100	0.99 ± 0.00	0.99 ± 0.00	0.99 ± 0.00	0.98 ± 0.00

**Table 4:** Fit Time ± SEM (seconds) of each method. Outcomes are simulated as Gaussian with exchangeable correlation. We indicate out-of-memory (30Gb) with symbol —.

N	$n_{i}$	One-step	GLS-Ex	GLS-Ind	Marginal	FoSR
25	5	20.66 ± 0.14	9.05 ± 0.07	8.05 ± 0.04	0.49 ± 0.00	0.16 ± 0.00
	25	23.24 ± 0.10	11.98 ± 0.04	11.99 ± 0.05	4.85 ± 0.03	0.27 ± 0.00
	100	54.87 ± 0.59	35.85 ± 0.55	35.03 ± 0.54	75.15 ± 1.07	1.17 ± 0.01
50	5	27.45 ± 0.18	16.00 ± 0.13	14.60 ± 0.11	1.15 ± 0.01	0.21 ± 0.00
	25	34.59 ± 0.14	21.37 ± 0.10	19.97 ± 0.13	29.79 ± 0.27	0.45 ± 0.00
	100	86.76 ± 1.05	64.70 ± 0.51	66.35 ± 0.63	415.64 ± 2.57	2.15 ± 0.01
100	5	39.33 ± 0.21	27.26 ± 0.13	26.51 ± 0.14	3.79 ± 0.03	0.31 ± 0.00
	25	73.25 ± 0.90	54.20 ± 0.73	51.83 ± 0.74	233.72 ± 1.69	1.08 ± 0.02
	100	146.26 ± 2.27	102.88 ± 1.43	87.89 ± 0.99	—	2.79 ± 0.01

**Table 5:** Functional Coefficient Estimation Performance (RMSE) relative to the FoSR fit $\left({\text{RMSE}}_{AR1}/{\text{RMSE}}_{\text{FoSR}}\right)$. Each cell contains the average of 300 replicates ± SEM (SEM= 0.00 indicates a value < 0.01). Table values below 1.0 indicate the one-step has more accurate coefficient estimates. Outcomes are simulated as binary with AR1 correlation coefficient ρ(s)=ρ∈{0.25,0.5,0.75}.

N	$n_{i}$	0.25	0.5	0.75
25	5	1.03 ± 0.01	1.02 ± 0.01	0.98 ± 0.01
	25	1.01 ± 0.01	0.99 ± 0.01	0.91 ± 0.01
	100	0.99 ± 0.00	0.97 ± 0.00	0.91 ± 0.01
50	5	1.02 ± 0.00	1.01 ± 0.01	0.98 ± 0.01
	25	1.00 ± 0.00	0.96 ± 0.00	0.90 ± 0.01
	100	0.98 ± 0.00	0.96 ± 0.00	0.91 ± 0.01
100	5	1.01 ± 0.00	1.00 ± 0.01	0.97 ± 0.01
	25	0.99 ± 0.00	0.97 ± 0.00	0.92 ± 0.01
	100	0.99 ± 0.00	0.97 ± 0.00	0.93 ± 0.00

**Table 6:** Functional Coefficient Pointwise 95% CI Coverage comparing FoSR with the one-step. Each cell contains the average of 300 replicates ± SEM (SEM= 0.00 indicates a value <0.01). Outcomes are simulated as binary with AR1 correlation coefficient ρ(s)=ρ∈{0.25,0.5,0.75}.

N	$n_{i}$	One-step			FoSR		
		0.25	0.5	0.75	0.25	0.5	0.75
25	5	0.89 ± 0.00	0.89 ± 0.00	0.88 ± 0.00	0.94 ± 0.00	0.90 ± 0.00	0.84 ± 0.00
	25	0.89 ± 0.00	0.89 ± 0.00	0.89 ± 0.00	0.92 ± 0.00	0.87 ± 0.00	0.75 ± 0.00
	100	0.90 ± 0.00	0.90 ± 0.00	0.90 ± 0.00	0.91 ± 0.00	0.85 ± 0.00	0.72 ± 0.00
50	5	0.90 ± 0.00	0.90 ± 0.00	0.90 ± 0.00	0.93 ± 0.00	0.90 ± 0.00	0.84 ± 0.00
	25	0.91 ± 0.00	0.91 ± 0.00	0.91 ± 0.00	0.92 ± 0.00	0.86 ± 0.00	0.74 ± 0.00
	100	0.91 ± 0.00	0.91 ± 0.00	0.90 ± 0.00	0.90 ± 0.00	0.84 ± 0.00	0.71 ± 0.00
100	5	0.92 ± 0.00	0.92 ± 0.00	0.92 ± 0.00	0.94 ± 0.00	0.90 ± 0.00	0.84 ± 0.00
	25	0.91 ± 0.00	0.91 ± 0.00	0.91 ± 0.00	0.91 ± 0.00	0.86 ± 0.00	0.74 ± 0.00
	100	0.89 ± 0.00	0.90 ± 0.00	0.90 ± 0.00	0.88 ± 0.00	0.82 ± 0.00	0.69 ± 0.00

**Table 7:** Functional Coefficient Joint 95% CI Coverage comparing FoSR with the one-step. Each cell contains the average of 300 replicates ± SEM (SEM= 0.00 indicates a value < 0.01). Outcomes are simulated as binary with AR1 correlation coefficient ρ(s)=ρ∈{0.25,0.5,0.75}.

N	$n_{i}$	One-step			FoSR		
		0.25	0.5	0.75	0.25	0.5	0.75
25	5	0.97 ± 0.00	0.97 ± 0.00	0.97 ± 0.00	0.99 ± 0.00	0.98 ± 0.00	0.95 ± 0.00
	25	0.98 ± 0.00	0.97 ± 0.00	0.97 ± 0.00	0.99 ± 0.00	0.97 ± 0.00	0.90 ± 0.00
	100	0.98 ± 0.00	0.98 ± 0.00	0.97 ± 0.00	0.98 ± 0.00	0.96 ± 0.00	0.88 ± 0.00
50	5	0.98 ± 0.00	0.98 ± 0.00	0.98 ± 0.00	0.99 ± 0.00	0.98 ± 0.00	0.95 ± 0.00
	25	0.98 ± 0.00	0.98 ± 0.00	0.98 ± 0.00	0.99 ± 0.00	0.97 ± 0.00	0.90 ± 0.00
	100	0.98 ± 0.00	0.98 ± 0.00	0.98 ± 0.00	0.98 ± 0.00	0.96 ± 0.00	0.87 ± 0.00
100	5	0.99 ± 0.00	0.98 ± 0.00	0.98 ± 0.00	0.99 ± 0.00	0.98 ± 0.00	0.95 ± 0.00
	25	0.98 ± 0.00	0.98 ± 0.00	0.98 ± 0.00	0.98 ± 0.00	0.96 ± 0.00	0.89 ± 0.00
	100	0.97 ± 0.00	0.97 ± 0.00	0.97 ± 0.00	0.97 ± 0.00	0.95 ± 0.00	0.86 ± 0.00

**Table 8:** Fit Time ±SEM (seconds) for the entire one-step estimation procedure. Outcomes are simulated as binary with AR1 correlation coefficient ρ(s)=ρ∈{0.25,0.5,0.75}.

N	$n_{i}$	0.25	0.5	0.75
25	5	20.78 ± 0.11	19.97 ± 0.17	19.01 ± 0.06
	25	26.69 ± 0.23	25.07 ± 0.23	23.45 ± 0.09
	100	49.67 ± 0.64	67.62 ± 0.50	46.37 ± 0.36
50	5	29.64 ± 0.11	29.27 ± 0.09	29.34 ± 0.10
	25	43.93 ± 0.25	39.98 ± 0.22	39.71 ± 0.20
	100	111.59 ± 1.42	98.36 ± 1.22	96.62 ± 1.02
100	5	49.28 ± 0.12	49.78 ± 0.17	49.33 ± 0.13
	25	73.97 ± 0.52	77.71 ± 0.70	73.89 ± 0.47
	100	212.35 ± 3.29	222.63 ± 2.92	170.01 ± 1.48

## Footnotes

1 Our implementation also allows for modeling covariance in the functional direction (instead of modeling covariance in the longitudinal direction) by adopting an alternative block diagonal correlation structure that specifies $\text{Cov}\left(Y_{i,j}(s),Y_{i,j}\left(s^{\prime }\right)\right)\neq 0$ and $\text{Cov}\left(Y_{i,j}(s),Y_{i,j^{\prime }}\left(s^{\prime }\right)\right)=0$ for $\left(s\neq s^{\prime }\right.$ and $\left.j\neq j^{\prime }\right)$.

2 Restricted Maximum Likelihood cannot be used to select smoothing parameters for fGEE because there is no likelihood to maximize (Chen et al., 2013).

3 The Marginal approach does not provide code for joint CIs.

## References

[R1] Allévius B. (2018). On the precision matrix of an irregularly sampled ar(1) process.

[R2] Ammar G. S. and Gragg W. B. (1988). Superfast solution of real positive definite toeplitz systems. SIAM Journal on Matrix Analysis and Applications, 9(1):61–76.

[R3] Barrett T., Dowle M., Srinivasan A., Gorecki J., Chirico M., Hocking T., Schwendinger B., and Krylov I. (2025). data.table: Extension of ‘data.frame’. R package version 1.17.99, https://Rdatatable.gitlab.io/data.table, https://github.com/Rdatatable/data.table.

[R4] Brockhaus S., Scheipl F., Hothorn T., and Greven S. (2015). The functional linear array model. Statistical Modelling, 15(3):279–300.

[R5] Chen H., Wang Y., Paik M. C., and Choi H. A. (2013). A marginal approach to reduced-rank penalized spline smoothing with application to multilevel functional data. Journal of the American Statistical Association, 108(504):1216–1229. 24497670 10.1080/01621459.2013.826134 PMC3909538

[R6] Cheng G., Yu Z., and Huang J. Z. (2013). The cluster bootstrap consistency in generalized estimating equations. Journal of Multivariate Analysis, 115:33–47.

[R7] Coddington L. T., Lindo S. E., and Dudman J. T. (2023). Mesolimbic dopamine adapts the rate of learning from action. Nature, 614(7947):294–302. 36653450 10.1038/s41586-022-05614-z PMC9908546

[R8] Crainiceanu C. M., Goldsmith J., Leroux A., and Cui E. (2024). Functional data analysis with R. CRC Press.

[R9] Cui E., Leroux A., Smirnova E., and Crainiceanu C. M. (2022). Fast univariate inference for longitudinal functional models. Journal of Computational and Graphical Statistics, 31(1):219–230. 35712524 10.1080/10618600.2021.1950006 PMC9197085

[R10] Degras D. (2017). Simultaneous confidence bands for the mean of functional data. WIREs Computational Statistics, 9(3):e1397.

[R11] Eckardt M., Mateu J., and Greven S. (2024). Generalized functional additive mixed models with (functional) compositional covariates for areal covid-19 incidence curves. Journal of the Royal Statistical Society Series C: Applied Statistics, 73(4):880–901.

[R12] Glaser J., Whiteway M., Cunningham J. P., Paninski L., and Linderman S. (2020). Recurrent switching dynamical systems models for multiple interacting neural populations. In Larochelle H., Ranzato M., Hadsell R., Balcan M., and Lin H., editors, Advances in Neural Information Processing Systems, volume 33, pages 14867–14878. Curran Associates, Inc.

[R13] Goldsmith J., Scheipl F., Huang L., Wrobel J., Di C., Gellar J., Harezlak J., McLean M. W., Swihart B., Xiao L., Crainiceanu C., Reiss P. T., and Cui E. (2024). refund regression with functional data. R package version 0.1–37.

[R14] Greven S., Crainiceanu C., Caffo B., and Reich D. (2011). Longitudinal functional principal component analysis. In Recent advances in functional data analysis and related topics, pages 149–154. Springer.

[R15] Grienberger C., Giovannucci A., Zeiger W., and Portera-Cailliau C. (2022). Two-photon calcium imaging of neuronal activity. Nature Reviews Methods Primers, 2(1):67. 10.1038/s43586-022-00147-1 PMC10732251 38124998

[R16] Guha Niyogi P. and Zhong P.-S. (2025). Quadratic inference with dense functional responses. Journal of Multivariate Analysis, 207:105400. 40766879 10.1016/j.jmva.2024.105400 PMC12320752

[R17] Guo W. (2002). Functional mixed effects models. Biometrics, 58(1):121–128. 11890306 10.1111/j.0006-341x.2002.00121.x

[R18] Inácio A. R., Lam K. C., Zhao Y., Pereira F., Gerfen C. R., and Lee S. (2025). Brain-wide presynaptic networks of functionally distinct cortical neurons. Nature. 10.1038/s41586-025-08631-w PMC12043506 40011781

[R19] Jeong H., Taylor A., Floeder J. R., Lohmann M., Mihalas S., Wu B., Zhou M., Burke D. A., and Namboodiri V. M. K. (2022). Mesolimbic dopamine release conveys causal associations. Science, 378(6626):eabq6740. 10.1126/science.abq6740 PMC9910357 36480599

[R20] Jewell S. and Witten D. (2018). Exact spike train inference via l0 optimization. The annals of applied statistics, 12(4):2457. 30627301 10.1214/18-AOAS1162 PMC6322847

[R21] Jun J. J., Steinmetz N. A., Siegle J. H., Denman D. J., Bauza M., Barbarits B., Lee A. K., Anastassiou C. A., Andrei A., Aydın Ç., Barbic M., Blanche T. J., Bonin V., Couto J., Dutta B., Gratiy S. L., Gutnisky D. A., Häusser M., Karsh B., Ledochowitsch P., Lopez C. M., Mitelut C., Musa S., Okun M., Pachitariu M., Putzeys J., Rich P. D., Rossant C., Sun W. l., Svoboda K., Carandini M., Harris K. D., Koch C., O’Keefe J., and Harris T. D. (2017). Fully integrated silicon probes for high-density recording of neural activity. Nature, 551(7679):232–236. 29120427 10.1038/nature24636 PMC5955206

[R22] Kuschnig N. (2023). sanic: Solving Ax = b Nimbly in C++. R package version 0.0.2.

[R23] Legaria A. A., Matikainen-Ankney B. A., Yang B., Ahanonu B., Licholai J. A., Parker J. G., and Kravitz A. V. (2022). Fiber photometry in striatum reflects primarily nonsomatic changes in calcium. Nature Neuroscience, 25(9):1124–1128. 36042311 10.1038/s41593-022-01152-z PMC10152879

[R24] Li R., Xiao L., Smirnova E., Cui E., Leroux A., and Crainiceanu C. M. (2022). Fixed-effects inference and tests of correlation for longitudinal functional data. Statistics in Medicine, 41(17):3349–3364. 35491388 10.1002/sim.9421 PMC9283332

[R25] Liang K.-Y. and Zeger S. L. (1986). Longitudinal data analysis using generalized linear models. Biometrika, 73(1):13–22.

[R26] Ling Y. and Lysy M. (2022). SuperGauss: Superfast Likelihood Inference for Stationary Gaussian Time Series. R package version 2.0.3.

[R27] Lipsitz S., Fitzmaurice G., Sinha D., Hevelone N., Hu J., and Nguyen L. L. (2017). One-step generalized estimating equations with large cluster sizes. Journal of Computational and Graphical Statistics, 26(3):734–737. 29422762 10.1080/10618600.2017.1321552 PMC5800532

[R28] Loewinger G., Cui E., Lovinger D., and Pereira F. (2025). A statistical framework for analysis of trial-level temporal dynamics in fiber photometry experiments. Elife, 13:RP95802. 10.7554/eLife.95802 PMC11903033 40073228

[R29] Molenberghs G., Verbeke G., (2005). Models for discrete longitudinal data.

[R30] Morris J. S. and Carroll R. J. (2006). Wavelet-based functional mixed models. Journal of the Royal Statistical Society Series B: Statistical Methodology, 68(2):179–199. 19759841 10.1111/j.1467-9868.2006.00539.x PMC2744105

[R31] Papadakis M., Tsagris M., Dimitriadis M., Fafalios S., Tsamardinos I., Fasiolo M., Borboudakis G., Burkardt J., Zou C., and Lakiotaki K. (2018). Package ‘rfast’.

[R32] Pnevmatikakis E. A., Soudry D., Gao Y., Machado T. A., Merel J., Pfau D., Reardon T., Mu Y., Lacefield C., Yang W., (2016). Simultaneous denoising, deconvolution, and demixing of calcium imaging data. Neuron, 89(2):285–299. 26774160 10.1016/j.neuron.2015.11.037 PMC4881387

[R33] Qu A. and Li R. (2005). Quadratic inference functions for varying-coefficient models with longitudinal data. Biometrics, 62(2):379–391. 10.1111/j.1541-0420.2005.00490.x PMC2680010 16918902

[R34] Roesch M. R., Singh T., Brown P. L., Mullins S. E., and Schoenbaum G. (2009). Ventral striatal neurons encode the value of the chosen action in rats deciding between differently delayed or sized rewards. Journal of Neuroscience, 29(42):13365–13376. 19846724 10.1523/JNEUROSCI.2572-09.2009 PMC2788608

[R35] Scheipl F., Gertheiss J., and Greven S. (2016). Generalized functional additive mixed models. Electronic Journal of Statistics. 10.1080/10618600.2014.901914 PMC4560367 26347592

[R36] Scheipl F., Staicu A.-M., and Greven S. (2015). Functional additive mixed models. Journal of Computational and Graphical Statistics, 24(2):477–501. 26347592 10.1080/10618600.2014.901914 PMC4560367

[R37] Sergazinov R., Leroux A., Cui E., Crainiceanu C., Aurora R. N., Punjabi N. M., and Gaynanova I. (2023). A case study of glucose levels during sleep using multilevel fast function on scalar regression inference. Biometrics, 79(4):3873–3882. 37189239 10.1111/biom.13878

[R38] Shou H., Zipunnikov V., Crainiceanu C. M., and Greven S. (2015). Structured functional principal component analysis. Biometrics, 71(1):247–257. 25327216 10.1111/biom.12236 PMC4383722

[R39] Touloumis A. (2016). Simulating correlated binary and multinomial responses under marginal model specification: The simcormultres package. The R Journal, 8:79–91. https://rjournal.github.io/.

[R40] Venables W. N. and Ripley B. D. (2002). Modern Applied Statistics with S. Springer, New York, fourth edition. ISBN 0-387-95457-0.

[R41] Walker G. T. (1931). On periodicity in series of related terms. Proceedings of the Royal Society of London. Series A, Containing Papers of a Mathematical and Physical Character, 131(818):518–532.

[R42] Wang X., Kolar M., and and A. S. (2025). Statistical inference for networks of high-dimensional point processes. Journal of the American Statistical Association, 120(550):1014–1024.

[R43] Willmore L., Minerva A. R., Engelhard B., Murugan M., McMannon B., Oak N., Thiberge S. Y., Peña C. J., and Witten I. B. (2023). Overlapping representations of food and social stimuli in mouse vta dopamine neurons. Neuron, 111(22):3541–3553.e8. 37657441 10.1016/j.neuron.2023.08.003 PMC11672631

[R44] Wood S. N. (2011). Fast stable restricted maximum likelihood and marginal likelihood estimation of semiparametric generalized linear models. Journal of the Royal Statistical Society Series B: Statistical Methodology, 73(1):3–36.

[R45] Wood S. N. (2017). Generalized Additive Models: An Introduction with R. Chapman and Hall/CRC, 2 edition.

[R46] Xia L. and Shojaie A. (2024). Inference for linear functionals of high-dimensional longitudinal proteomics data using generalized estimating equations.

[R47] Yule G. U. (1927). Vii. on a method of investigating periodicities disturbed series, with special reference to wolfer’s sunspot numbers. Philosophical Transactions of the Royal Society of London. Series A, Containing Papers of a Mathematical or Physical Character, 226(636–646):267–298.

[R48] Zhang Y., Rózsa M., Liang Y., Bushey D., Wei Z., Zheng J., Reep D., Broussard G. J., Tsang A., Tsegaye G., Narayan S., Obara C. J., Lim J.-X., Patel R., Zhang R., Ahrens M. B., Turner G. C., Wang S. S. H., Korff W. L., Schreiter E. R., Svoboda K., Hasseman J. P., Kolb I., and Looger L. L. (2023). Fast and sensitive gcamp calcium indicators for imaging neural populations. Nature, 615(7954):884–891. 36922596 10.1038/s41586-023-05828-9 PMC10060165

[R49] Zhou X., Cui E., Sartini J., and Crainiceanu C. (2025). Prediction inference using generalized functional mixed effects models. arXiv preprint arXiv:2501.07842.

[R50] Zhu H., Chen K., Luo X., Yuan Y., and Wang J.-L. (2019). Fmem: Functional mixed effects models for longitudinal functional responses. Statistica Sinica, 29(4):2007. 31745381 10.5705/ss.202017.0505 PMC6863349

[R51] Zipunnikov V., Greven S., Shou H., Caffo B., Reich D. S., and Crainiceanu C. (2014). Longitudinal high-dimensional principal components analysis with application to diffusion tensor imaging of multiple sclerosis. The annals of applied statistics, 8(4):2175. 25663955 10.1214/14-aoas748 PMC4316386

