# Supplementary material for: Fast Penalized Generalized Estimating Equations for Large Longitudinal Functional Datasets
Source: ArXiv. 2025 Jun 25:arXiv:2506.20437v1. Preprint. [Version 1] (PMC12306803)
Supplement: 1 [file NIHPP2506.20437V1-supplement-1.pdf]

# Appendix A Additional Method Details

## A.1 Cross-Validation Procedure

### A.1.1 Full-Sample Cross Validation

We define a standard cluster K-Fold Cross-Validation One-Step estimator as

$$\widehat{\boldsymbol{\theta}}^{k}_{\Lambda_1} = \widehat{\boldsymbol{\theta}}^{(0)}_{\Lambda_0} + \frac{1}{N - |\mathcal{K}_k|}\left[\frac{1}{N - |\mathcal{K}_k|}\sum_{i \notin \mathcal{K}_k} \mathbb{W}_i(\widehat{\boldsymbol{\theta}}^{(0)}_{\Lambda_0}) + n^*_k \Lambda_1 \mathbb{S}\right]^{-1} \sum_{i \notin \mathcal{K}_k}\left\{\mathbb{B}_i(\widehat{\boldsymbol{\theta}}^{(0)}_{\Lambda_0}) - n^*_k \Lambda_1 \mathbb{S} \widehat{\boldsymbol{\theta}}^{(0)}_{\Lambda_0}\right\}, \tag{10}$$

where $n^*_k = \frac{\sum_{i \notin \mathcal{K}_k} n_i}{\sum_{i=1}^{N} n_i}$.

### A.1.2 Sequential Tuning Procedure

The diagonal smoothing matrix $\Lambda$ contains smoothing parameters $\lambda_1, \ldots, \lambda_q$, each repeated based on the number of knots used for its functional coefficient. To identify the correct range for the $\lambda_1, \ldots, \lambda_q$, we apply an iterative CV strategy that is designed to be fast as $q$ grows. By dividing the tuning into three stages, we avoid tuning over a large $q$ dimensional grid that can be computationally impractical even for $q \geq 3$. In step (1), we tune over a small one-dimensional grid to identify the correct order of magnitude for the smoothing parameters: $\boldsymbol{\Lambda}_{(1)} = \{\alpha_1 \Lambda^{(0)}, \ldots, \alpha_L \Lambda^{(0)}\}$ where, for example, $\{\alpha_1, \ldots, \alpha_{L_1}\} = \{0.001, 0.01, 0.1, 1, 10, 100, 1000\}$. This exploits the fact that the unique diagonal entries $\lambda^{(0)}_1, \ldots, \lambda^{(0)}_q$ of the $\Lambda_0$ selected by REML are, in our experience, on a reasonable relative scale. In step (2), we tune over a small $q$-dimensional grid constructed around the smoothing parameter values that minimize the cross-validated criteria (e.g. MSE, negative log-likelihood) in step (1). Denoting $\tilde{\lambda}^{(1)}_1, \ldots, \tilde{\lambda}^{(1)}_q$ as these selected values, we tune over a grid with all unique combinations (with the `R` function `expand.grid()`) of grid $\boldsymbol{\Lambda}_{(2)} = \left\{\{\alpha_1 \tilde{\lambda}^{(1)}_1, \ldots, \alpha_{L_2} \tilde{\lambda}^{(1)}_1\}, \ldots, \{\alpha_1 \tilde{\lambda}^{(1)}_q, \ldots, \alpha_L \tilde{\lambda}^{(1)}_q\}\right\}$, where, for example, $\{\alpha_1, \ldots, \alpha_{L_2}\} =$
$\{0.001, 0.01, 0.1, 1, 10, 100, 1000\}$. Step (2) identifies a reasonable order of magnitude for each smoothing parameter with a small grid. Finally, in step (3), we tune each smoothing parameter within a local neighborhood around the selected values from step (2), $\tilde{\lambda}^{(2)}_1, \ldots, \tilde{\lambda}^{(2)}_q$. Specifically,

we tune over the unique combinations, $\boldsymbol{\Lambda}_{(3)} = \Big\{\{\alpha_1\tilde{\lambda}_1^{(2)}, \dots, \alpha_{L_3}\tilde{\lambda}_1^{(2)}\}, \dots, \{\alpha_1\tilde{\lambda}_q^{(2)}, \dots, \alpha_{L_3}\tilde{\lambda}_q^{(2)}\}\Big\}$, where, for example, $\{\alpha_1, \dots, \alpha_{L_3}\} \subset [0.1, 10]$. We found this was fast and performed well in simulations for both continuous and binary outcomes, across a wide range of $N$ and $n_i$ values. We anticipate the relative speedups of using fast K-fold CV, compared to standard K-fold CV, would be grow as $p$ increases (e.g. from increasing the number of knots or covariates, $q$). This is because fast K-fold inverts a $p \times p$ matrix only once per $\Lambda_1$ value, whereas standard K-fold inverts a similar $p \times p$ matrix for every unique $\{k, \Lambda_1\}$ pair.

### A.1.3 Cross-Validation Strategy Performance Comparison

Here we compare performance of the One-Step Estimator trained with the "fast k-fold CV" vs. standard "k-fold CV." The fast k-fold uses the full-sample matrix $\left[\frac{1}{N}\left(\sum_{i=1}^{N} \nabla_{\boldsymbol{\theta}} \widetilde{\boldsymbol{U}}_{\boldsymbol{\theta}}(\widehat{\boldsymbol{\theta}}_{\Lambda_0}^{(0)})\right) + \Lambda\mathbb{S}\right]^{-1}$ and keeps that fixed across folds, whereas the "k-fold CV" uses $\left[\frac{1}{N-|\mathcal{K}_k|}\sum_{i\notin\mathcal{K}_k} \mathbb{W}_i(\widehat{\boldsymbol{\theta}}_{\Lambda_0}^{(0)}) + n_k^*\Lambda_1\mathbb{S}\right]^{-1}$ with the appropriate scaling. We set $K = 10$. These results are from the simulation scheme described in main text Section 4.2 "Simulation 2: Gaussian Outcome with Exchangeable Correlation." Table 9 shows the RMSE of the functional coefficients relative the initial FoSR fit $\widehat{\boldsymbol{\beta}}_{\Lambda_0}^{(0)}$ (fit with `pffr`). The performance of the "fast k-fold CV" is equal or better than that of "k-fold CV." Table 10 shows the pointwise CI coverage on the One-Step after selecting $\Lambda_1$ with the two CV procedures. It shows the coverage is indistinguishable. Finally Table 11 shows the runtime, where Fast K-Fold is usually considerably faster (though this depends on $N$ and $n_i$).

| $N$ | $n_i$ | Fast K-fold | K-fold |
|---|---|---|---|
| 25 | 5 | 0.96 ± 0.00 | 0.96 ± 0.00 |
| | 25 | 0.99 ± 0.00 | 0.99 ± 0.00 |
| | 100 | 1.01 ± 0.00 | 1.01 ± 0.00 |
| 50 | 5 | 0.95 ± 0.00 | 0.96 ± 0.00 |
| | 25 | 0.98 ± 0.00 | 0.98 ± 0.00 |
| | 100 | 1.00 ± 0.00 | 1.01 ± 0.00 |
| 100 | 5 | 0.95 ± 0.00 | 0.95 ± 0.00 |
| | 25 | 0.98 ± 0.00 | 0.98 ± 0.00 |
| | 100 | 0.99 ± 0.00 | 0.99 ± 0.00 |

Table 9: Comparison of Cross-Validation methods. One-Step Functional Coefficient Estimation Performance (RMSE), tuned with 'Fast K-Fold' or 'K-Fold CV', relative to the initial FoSR fit $\widehat{\boldsymbol{\beta}}^{(0)}_{\Lambda_0}$: (RMSE/RMSE$_{FoSR}$). Cells contain the average of 300 replicates ± SEM (SEM= 0.00 indicates a value $< 0.01$). The results indicate that the "Fast" version of K-Fold CV results in comparable coefficient estimation accuracy to the standard version ($K = 10$).

| $N$ | $n_i$ | Fast K-fold | K-fold |
|---|---|---|---|
| 25 | 5 | 0.95 ± 0.00 | 0.95 ± 0.00 |
| | 25 | 0.95 ± 0.00 | 0.95 ± 0.00 |
| | 100 | 0.92 ± 0.00 | 0.92 ± 0.00 |
| 50 | 5 | 0.96 ± 0.00 | 0.96 ± 0.00 |
| | 25 | 0.97 ± 0.00 | 0.97 ± 0.00 |
| | 100 | 0.92 ± 0.00 | 0.92 ± 0.00 |
| 100 | 5 | 0.96 ± 0.00 | 0.96 ± 0.00 |
| | 25 | 0.97 ± 0.00 | 0.97 ± 0.00 |
| | 100 | 0.97 ± 0.00 | 0.97 ± 0.00 |

Table 10: Comparison of Cross-Validation methods: Pointwise 95% CI coverage of the final One-Step fit after selecting $\Lambda_1$ with Fast K-Fold or K-Fold CV. $K = 10$. Cells contain the average of 300 replicates ± SEM (SEM= 0.00 indicates a value $< 0.01$).

| $N$ | $n_i$ | Fast K-fold | K-fold |
|---|---|---|---|
| 25 | 5 | 5.92 ± 0.16 | 12.32 ± 0.30 |
| | 25 | 8.73 ± 0.19 | 18.55 ± 0.44 |
| | 100 | 28.77 ± 0.60 | 44.42 ± 0.81 |
| 50 | 5 | 9.12 ± 0.23 | 15.40 ± 0.37 |
| | 25 | 19.12 ± 0.36 | 26.01 ± 0.49 |
| | 100 | 108.89 ± 4.01 | 108.86 ± 3.62 |
| 100 | 5 | 15.56 ± 0.35 | 22.76 ± 0.54 |
| | 25 | 83.58 ± 2.54 | 74.23 ± 2.42 |
| | 100 | 93.37 ± 2.03 | 78.04 ± 1.67 |

Table 11: Comparison of Cross-Validation methods: Entire Fitting Time of Entire One-Step Estimator with Fast K-Fold or K-Fold CV. $K = 10$. Cells contain the average of 300 replicates ± SEM.

## A.2 Penalized Generalized Least Squares

The penalized Generalized Least Squares (GLS) approach used as a benchmark in the simulations is calculated with the closed-form estimator

$$\widehat{\boldsymbol{\theta}}_{\Lambda_G}^{GLS} = \frac{1}{N}\left[\frac{1}{N}\left(\sum_{i=1}^{N}\mathbb{X}_i^T[\widehat{\mathbb{V}}_i(\widehat{\boldsymbol{\theta}}_{\Lambda_0}^{(0)})]^{-1}\mathbb{X}_i\right)+\Lambda_G\mathbb{S}\right]^{-1}\sum_{i=1}^{N}\mathbb{X}_i^T[\widehat{\mathbb{V}}_i(\widehat{\boldsymbol{\theta}}_{\Lambda_0}^{(0)})]^{-1}\boldsymbol{Y}_i \tag{11}$$

where $\Lambda_0$ is the smoothing parameters selected (with fast restricted maximum likelihood) in the initial fit, $\widehat{\boldsymbol{\theta}}_{\Lambda_0}^{(0)}$, used in the One-Step estimator. We use the notation $\Lambda_G$ to denote the smoothing parameters selected based on a GLS-specific fast K-fold cross-validation (CV) procedure. This CV approach is identical to the One-Step fast K-fold CV procedure, except that coefficients are estimated with the above GLS closed-form expression instead of the One-Step estimator. For the exchangeable GLS (*GLS-Ex*), and AR1 GLS (*GLS-AR1*) estimators, we construct $\mathbb{V}_i$ with the same form as used for the corresponding One-Step $\mathbb{V}_i$. For the independent correlation structure GLS (*GLS-Ind*), we set $\mathbb{V}_i(s) = \mathbf{A}_i^{1/2}(s)\mathbf{R}_i(s)\mathbf{A}_i^{1/2}(s) = \mathbf{A}_i^{1/2}(s)\mathbf{I}_{n_i}\mathbf{A}_i^{1/2}(s) = \mathbf{A}_i(s)$, where $\mathbf{A}_i(s) = \mathrm{diag}\left(v_{i,1}(s), \ldots, v_{i,n_i}(S)\right)$ and $v_{i,j}(s) = \mathrm{Var}(Y_{i,j}(s) \mid \mathbb{X}_{i,j})$. Otherwise, smoothing parameter tuning, coefficient estimation, and variance calculation is identical to the other GLS estimators.

We estimate $\mathrm{Var}\left(\widehat{\boldsymbol{\theta}}_{\Lambda_G}^{GLS}\right)$ with the same sandwich estimator as the One-Step

$$\mathrm{Var}(\widehat{\boldsymbol{\theta}}_{\Lambda}^{GLS}) = \boldsymbol{H}_{\Lambda}^{-1}\boldsymbol{M}\boldsymbol{H}_{\Lambda}^{-1}, \tag{12}$$

where $\boldsymbol{H}_{\Lambda}^{-1} = \sum_{i=1}^{N}\mathbb{X}_i^T\mathbb{V}_i^{-1}\mathbb{X} + \Lambda\mathbb{S}$, and
$\boldsymbol{M} = \sum_{i=1}^{N}\mathbb{X}_i^T\mathbf{A}_i\mathbb{V}_i^{-1}[\boldsymbol{Y}_i - g^{-1}(\mathbb{X}_i\boldsymbol{\theta})][\boldsymbol{Y}_i - g^{-1}(\mathbb{X}_i\boldsymbol{\theta})]^T\mathbb{V}_i^{-1}\mathbf{A}_i\mathbb{X}_i$, but we instead plug in $\widehat{\boldsymbol{\theta}}_{\Lambda_G}^{GLS}$, $\widehat{\mathbb{V}}_i(\widehat{\boldsymbol{\theta}}_{\Lambda_G}^{GLS})$, $\widehat{\mathbf{A}}_i(\widehat{\boldsymbol{\theta}}_{\Lambda_G}^{GLS})$, and $\Lambda_G$. For example, to calculate $\widehat{\mathbb{V}}_i(\widehat{\boldsymbol{\theta}}_{\Lambda_G}^{GLS})$, we estimate the correlation parameters, $\widehat{\rho}(s)$, using the GLS estimates, $\widehat{\boldsymbol{\theta}}_{\Lambda_G}^{GLS}$.

## A.3 Initial FoSR `pffr` fit

As an initial fit, the penalized maximum likelihood estimator

$$\widehat{\boldsymbol{\theta}}_{\Lambda_0}^{(0)} = \underset{\boldsymbol{\theta}}{\text{argmin}} \quad -2\sum_{i=1}^{N}\sum_{j=1}^{n_i}\sum_{s\in\mathcal{S}} l(Y_{i,j}(s), \mathbb{X}_{i,j}; \boldsymbol{\theta}) + \boldsymbol{\theta}^T \Lambda_0 \mathbb{S} \boldsymbol{\theta}, \tag{13}$$

where $l(Y_{i,j}(s), \mathbb{X}_{i,j}; \boldsymbol{\theta})$ is the log-likelihood evaluated on a single observation of the outcome $Y_{i,j}(s) \in \mathbb{R}$ from cluster $i$, at longitudinal observation $j$, at functional domain point $s$. We denote the model parameter $\boldsymbol{\theta}$, $\mathbb{S}$ as the penalty matrix, and $\Lambda_0$ as an associated diagonal matrix of smoothing parameters. As discussed in the main text, $\mathbb{X}_{i,j} \in \mathbb{R}^p$ is constructed as a product of pre-defined basis functions (e.g. B-splines) and the original covariates, $\boldsymbol{x}_{i,j} \in \mathbb{R}^q$. Model 13 is equivalent to adopting a correlation structure that assumes all observations $Y_{i,j}(s)$ are mutually independent across $i$, $j$, and $s$.

We fit model 13 with the `refund` package in `R` (Goldsmith et al., 2024) with the `pffr` function (Scheipl et al., 2015). This calls the `mgcv` package in `R` (Wood, 2017) to fit the model with the `gam` or `bam` functions. For example, for a model with two covariates, `X1` and `X2`, we use the following code:

```
initial_fit = refund::pffr(Y ~ X1 + X2,
                           family = fam,
                           algorithm = "bam",
                           method = "fREML",
                           discrete = TRUE,
                           bs.yindex = list(bs = spline.basis,
                                            k = knots,
                                            m = m.pffr),
                           data = data_df)
```

where `fam` is the exponential dispersion family adopted to construct a pseudo-likelihood based estimating equation in the fGEE, `spline.basis` is the pre-specified basis spline from the `mgcv` family (e.g. `bs`, `ps`, `tp`), `knots` is the number of knots, and `m.pffr` is the penalty type. For

example, as noted in the `pffr` function, the following `bs.yindex = list(bs="ps", k=5, m=c(2, 1))` indicates 5 cubic B-splines bases with a first order difference penalty.

### A.4 Computational Details

Our implementation uses a number of `R` packages for estimation of nuisance parameters and to improve computational speed. We use the `data.table` package extensively to increase computational efficiency (Barrett et al., 2025). Our code structure is loosely based on the structure from the `gee1step)` package that implements the One-Step (non-functional) GEE (Lipsitz et al., 2017) available on the `Github` `https://github.com/kgoldfeld/gee1step` of Professor Keith Goldfeld. We use the `Rfast` package (Papadakis et al., 2018) to estimate the $\rho(s)$ for AR1 correlation and to speed up other standard computations. We use the MASS package to draw multivariate normals (Venables and Ripley, 2002). We use Allévius (2018) to estimate and invert covariance matrices with an AR1 structure when the time intervals are irregular. We use the `SuperGauss` package (Ling and Lysy, 2022) to invert AR1 matrices with regular time intervals. We use the `sanic` package to quickly invert other positive definite matrices (Kuschnig, 2023). As mentioned in the main text and previous Appendix section, we use `mgcv` (Wood, 2017) and `refund` (Goldsmith et al., 2024) packages to estimate initial fits and to (optionally) smooth the correlation parameters across the functional domain.

## Appendix B Theory

Throughout, we fix an arbitrary matrix norm $\|\cdot\|$ (e.g., operator, Frobenius), and for a sequence of random matrices $(A_N)_{N=1}^{\infty}$, we write $A_N = O_{\mathbb{P}}(1)$ if $\|A_N\| = O_{\mathbb{P}}(1)$, and $A_N = o_{\mathbb{P}}(1)$ if $\|A_N\| = o_{\mathbb{P}}(1)$. For symmetric matrix $A$, we write $\lambda_{\min}(A)$ and $\lambda_{\max}(A)$ for the smallest and largest eigenvalues of $A$, respectively. We begin by stating and proving a lemma that we use to prove our general theorem.

**Lemma B.1.** *Let $A_N \in \mathbb{R}^{p\times p}$ be a sequence of fixed and invertible matrices such that $A_N = O(1)$ and $A_N^{-1} = O(1)$, let $X_N \in \mathbb{R}^p$ be a sequence of random vectors such that for constant scalars $r_N \to \infty$, $r_N A_N X_N = O_{\mathbb{P}}(1)$. If $B_N \in \mathbb{R}^{p\times p}$ is a set of random matrices that satisfy $B_N \xrightarrow{\mathbb{P}} \mathbf{0}_{p\times p}$, then $r_N A_N B_N X_N \xrightarrow{\mathbb{P}} \mathbf{0}_p$.*

*Proof.* Noting that $A_N B_N = O(1)o_{\mathbb{P}}(1) = o_{\mathbb{P}}(1)$, and

$$r_N X_N = A_N^{-1}(r_N A_N X_N) = O(1)O_{\mathbb{P}}(1) = O_{\mathbb{P}}(1)$$

we can immediately conclude that

$$r_N A_N B_N X_N = A_N B_N(r_N X_N) = o_{\mathbb{P}}(1)O_{\mathbb{P}}(1) = o_{\mathbb{P}}(1),$$

as claimed. □

We now consider a general adaptive $M$-estimation setting. Suppose we observe an iid sequence of random vectors $(Z_i)_{i=1}^{\infty}$, with generic observation denoted $Z \sim \mathbb{P}$, and for each fixed sample size $N$ we work with the differentiable (in $\boldsymbol{\theta}$) estimating equation $\boldsymbol{U}_N(Z;\boldsymbol{\theta}) \in \mathbb{R}^p$, for parameters $\boldsymbol{\theta} \in \mathbb{R}^p$. We write $U_{N,\ell}(Z;\boldsymbol{\theta})$ for the $\ell$-th component of $\boldsymbol{U}_N(Z;\boldsymbol{\theta})$. The "fully iterated" estimator $\widehat{\boldsymbol{\theta}}_N^*$ would be given by solving

$$\mathbb{P}_N\left\{\boldsymbol{U}_N(Z;\boldsymbol{\theta})\right\} \equiv \frac{1}{N}\sum_{i=1}^{N}\boldsymbol{U}_N(Z_i;\boldsymbol{\theta}) = \mathbf{0}_p,$$

targeting the population parameter $\boldsymbol{\theta}_N$ that solves $\mathbb{E}\{\boldsymbol{U}_N(Z;\boldsymbol{\theta})\} = \mathbf{0}_p$. In practice, we will apply the general theory to the longitudinal functional setup by taking $Z_i \equiv (\mathbb{X}_i, \boldsymbol{Y}_i)$ and $\boldsymbol{U}_N(Z_i;\boldsymbol{\theta}) = \mathbb{D}_i^T\widetilde{\mathbb{V}}_{i,n}^{-1}(\boldsymbol{Y}_i - g^{-1}(\mathbb{X}_i\boldsymbol{\theta})) - \frac{1}{N}\Lambda_N\mathbb{S}\boldsymbol{\theta}$. In this special case, so long as $\Lambda_N \to \mathbf{0}_{p\times p}$ and $\widetilde{\mathbb{V}}_n^{-1} \to \mathbb{V}^{-1}$, we have $\boldsymbol{U}_N \to \boldsymbol{U}_\infty$ where $\boldsymbol{U}_\infty(Z;\boldsymbol{\theta}) = \mathbb{D}^T\mathbb{V}^{-1}(\boldsymbol{Y} - g^{-1}(\mathbb{X}\boldsymbol{\theta}))$, with corresponding parameter $\boldsymbol{\theta}_\infty$ solving $\mathbb{E}\{\boldsymbol{U}_\infty(Z;\boldsymbol{\theta})\} = \mathbf{0}_p$—note that we do not explicitly require such convergence in our general setup.

We require notation for a number of related important quantities. First, define the variance quantities $\boldsymbol{H}_N(\boldsymbol{\theta}) = \mathbb{E}\{\nabla_{\boldsymbol{\theta}}\boldsymbol{U}_N(Z;\boldsymbol{\theta})\}$, $\boldsymbol{M}_N(\boldsymbol{\theta}) = \mathbb{E}\{\boldsymbol{U}_N(Z;\boldsymbol{\theta})\boldsymbol{U}_N(Z;\boldsymbol{\theta})^T\}$, and

$$\boldsymbol{V}_N(\boldsymbol{\theta}) = \frac{1}{N}\{\boldsymbol{H}_N(\boldsymbol{\theta})\}^{-1}\boldsymbol{M}_N(\boldsymbol{\theta})\{\boldsymbol{H}_N(\boldsymbol{\theta})\}^{-1}.$$

Next, for an initial estimator $\widehat{\boldsymbol{\theta}}_N^{(0)}$, we define

$$\widehat{\boldsymbol{\theta}}_N^{(1)} \coloneqq \widehat{\boldsymbol{\theta}}_N^{(0)} - \left(\mathbb{P}_N\left[\nabla_{\boldsymbol{\theta}}\boldsymbol{U}_N(Z;\boldsymbol{\theta})\Big|_{\boldsymbol{\theta}=\widehat{\boldsymbol{\theta}}_N^{(0)}}\right]\right)^{-1}\mathbb{P}_N\left[\boldsymbol{U}_N(Z;\widehat{\boldsymbol{\theta}}_N^{(0)})\right].$$

Under weak conditions, writing $\{\boldsymbol{V}_N(\boldsymbol{\theta}_N)\}^{-1/2} = \sqrt{N}\{\boldsymbol{M}_N(\boldsymbol{\theta}_N)\}^{-1/2}\boldsymbol{H}_N(\boldsymbol{\theta}_N)$, we typically have the following asymptotic normality result for the fully iterated estimator:

$$\{\boldsymbol{V}_N(\boldsymbol{\theta}_N)\}^{-1/2}\left(\widehat{\boldsymbol{\theta}}_N^{*} - \boldsymbol{\theta}_N\right) \xrightarrow{d} \mathcal{N}(\mathbf{0}_p, I_p).$$

In the following result, we lay out conditions under which the one-step estimator $\widehat{\boldsymbol{\theta}}_N^{(1)}$ achieves the same convergence properties, i.e., is asymptotically equivalent to $\widehat{\boldsymbol{\theta}}_N^{*}$.

**Theorem B.2.** *Suppose the following conditions hold:*

*(i)* $\boldsymbol{U}_N(z;\boldsymbol{\theta})$ *is twice differentiable in* $\boldsymbol{\theta}$ *for all* $z$*, and the second derivative is uniformly bounded:* $\exists\, C_1 > 0$ *such that* $\mathbb{P}\left[\sup_{\boldsymbol{\theta}}\left|\frac{\partial^2 U_{N,\ell}(Z;\boldsymbol{\theta})}{\partial\theta_j\partial\theta_k}\right| \le C_1\right] = 1$*, for all* $j,k,\ell$*.*

*(ii) The second and third moments of* $\boldsymbol{U}_N(Z;\boldsymbol{\theta}_N)$ *are uniformly bounded below and above, respectively:* $\exists\, s,t > 0$ *such that* $\lambda_{\min}(\boldsymbol{M}_N(\boldsymbol{\theta}_N)) \ge s$*, and*

$$\mathbb{E}(|U_{N,j}(Z;\boldsymbol{\theta}_N)U_{N,k}(Z;\boldsymbol{\theta}_N)U_{N,\ell}(Z;\boldsymbol{\theta}_N)|) \le t,$$

*for all* $N \in \mathbb{N}$ *and all* $j,k,\ell$*.*

*(iii)* $\boldsymbol{H}_N(\boldsymbol{\theta}_N) =$ *is invertible and* $\mathbb{P}\left[\mathbb{P}_N\left(\nabla_{\boldsymbol{\theta}}\boldsymbol{U}_N(Z;\boldsymbol{\theta})\Big|_{\boldsymbol{\theta}=\widehat{\boldsymbol{\theta}}_N^{(0)}}\right) \text{ is non-singular}\right] = 1$*, for all* $N \in \mathbb{N}$*. Moreover,* $\{\boldsymbol{H}_N(\boldsymbol{\theta}_N)\}^{-1} = O(1)$ *and* $\left(\mathbb{P}_N\left[\nabla_{\boldsymbol{\theta}}\boldsymbol{U}_N(Z;\boldsymbol{\theta})\Big|_{\boldsymbol{\theta}=\widehat{\boldsymbol{\theta}}_N^{(0)}}\right]\right)^{-1} = O_{\mathbb{P}}(1)$*.*

*(iv)* $\boldsymbol{M}_N(\boldsymbol{\theta}_N) = O(1)$ *and* $\boldsymbol{H}_N(\boldsymbol{\theta}_N) = O(1)$*.*

*(v)* $\exists\, C_2 > 0 : \mathbb{E}(\|\nabla_{\boldsymbol{\theta}}\boldsymbol{U}_N(Z;\boldsymbol{\theta})|_{\boldsymbol{\theta}=\boldsymbol{\theta}_N} - \boldsymbol{H}_N(\boldsymbol{\theta}_N)\|^2) \le C_2$*, for all* $N \in \mathbb{N}$*.*

*(vi)* $\sqrt{N}\{\boldsymbol{M}_N(\boldsymbol{\theta}_N)\}^{-1/2}\boldsymbol{H}_N(\boldsymbol{\theta}_N)\left(\widehat{\boldsymbol{\theta}}_N^{(0)} - \boldsymbol{\theta}_N\right) = O_{\mathbb{P}}(1)$*.*

*Then the one-step estimator satisfies* $\{\boldsymbol{V}_N(\boldsymbol{\theta}_N)\}^{-1/2}\left(\widehat{\boldsymbol{\theta}}_N^{(1)} - \boldsymbol{\theta}_N\right) \xrightarrow{d} \mathcal{N}(\mathbf{0}_p, I_p)$*.*

*Proof of Theorem B.2.* Writing $\boldsymbol{U}_N^{(N)}(\boldsymbol{\theta}) = \mathbb{P}_N\left[\boldsymbol{U}_N(Z;\boldsymbol{\theta})\right]$, and employing a Taylor expansion of $\boldsymbol{U}_N^{(N)}$ at the initial estimator around $\boldsymbol{\theta}_N$, we have

$$\boldsymbol{U}_N^{(N)}(\widehat{\boldsymbol{\theta}}_N^{(0)}) = \boldsymbol{U}_N^{(N)}(\boldsymbol{\theta}_N) + \nabla_{\boldsymbol{\theta}}\boldsymbol{U}_N^{(N)}(\boldsymbol{\theta})\Big|_{\boldsymbol{\theta}=\boldsymbol{\theta}_N}\left(\widehat{\boldsymbol{\theta}}_N^{(0)} - \boldsymbol{\theta}_N\right) + \begin{bmatrix} (\widehat{\boldsymbol{\theta}}_N^{(0)} - \boldsymbol{\theta}_N)^T\boldsymbol{Q}_{N,1}(\widetilde{\boldsymbol{\theta}}_N)(\widehat{\boldsymbol{\theta}}_N^{(0)} - \boldsymbol{\theta}_N) \\ \vdots \\ (\widehat{\boldsymbol{\theta}}_N^{(0)} - \boldsymbol{\theta}_N)^T\boldsymbol{Q}_{N,p}(\widetilde{\boldsymbol{\theta}}_N)(\widehat{\boldsymbol{\theta}}_N^{(0)} - \boldsymbol{\theta}_N), \end{bmatrix}$$

for some $\widetilde{\boldsymbol{\theta}}$ on the line segment between $\widehat{\boldsymbol{\theta}}_N^{(0)}$ and $\boldsymbol{\theta}_N$, and where $\boldsymbol{Q}_{N,j}(\boldsymbol{\theta}) = \nabla^2_{\boldsymbol{\theta}}\, U_{N,j}^{(N)}(\boldsymbol{\theta}) \in \mathbb{R}^{p\times p}$ for each $j \in [p]$. By definition of the one-step estimator,

$$\begin{aligned}\widehat{\boldsymbol{\theta}}_N^{(1)} - \boldsymbol{\theta}_N &= (\widehat{\boldsymbol{\theta}}_N^{(0)} - \boldsymbol{\theta}_N) - \left(\mathbb{P}_N\left[\nabla_{\boldsymbol{\theta}}\boldsymbol{U}_N(Z;\boldsymbol{\theta})\Big|_{\boldsymbol{\theta}=\widehat{\boldsymbol{\theta}}_N^{(0)}}\right]\right)^{-1}\mathbb{P}_N\left[\boldsymbol{U}_N(Z;\widehat{\boldsymbol{\theta}}_N^{(0)})\right] \\ &= (\widehat{\boldsymbol{\theta}}_N^{(0)} - \boldsymbol{\theta}_N) - \left(\nabla_{\boldsymbol{\theta}}\boldsymbol{U}_N^{(N)}(\boldsymbol{\theta})\Big|_{\boldsymbol{\theta}=\widehat{\boldsymbol{\theta}}_N^{(0)}}\right)^{-1}\boldsymbol{U}_N^{(N)}(\widehat{\boldsymbol{\theta}}_N^{(0)}),\end{aligned}$$

so the Taylor expansion implies

$$\begin{aligned}&\widehat{\boldsymbol{\theta}}_N^{(1)} - \boldsymbol{\theta}_N \\ &= -\left(\nabla_{\boldsymbol{\theta}}\boldsymbol{U}_N^{(N)}(\boldsymbol{\theta})\Big|_{\boldsymbol{\theta}=\widehat{\boldsymbol{\theta}}_N^{(0)}}\right)^{-1}\boldsymbol{U}_N^{(N)}(\boldsymbol{\theta}_N) + \Bigg\{I_p - \left(\nabla_{\boldsymbol{\theta}}\boldsymbol{U}_N^{(N)}(\boldsymbol{\theta})\Big|_{\boldsymbol{\theta}=\widehat{\boldsymbol{\theta}}_N^{(0)}}\right)^{-1}\nabla_{\boldsymbol{\theta}}\boldsymbol{U}_N^{(N)}(\boldsymbol{\theta})\Big|_{\boldsymbol{\theta}=\boldsymbol{\theta}_N} \\ &\qquad - \left(\nabla_{\boldsymbol{\theta}}\boldsymbol{U}_N^{(N)}(\boldsymbol{\theta})\Big|_{\boldsymbol{\theta}=\widehat{\boldsymbol{\theta}}_N^{(0)}}\right)^{-1}\begin{bmatrix}(\widehat{\boldsymbol{\theta}}_N^{(0)} - \boldsymbol{\theta}_N)^T\boldsymbol{Q}_{N,1}(\widetilde{\boldsymbol{\theta}}_N) \\ \vdots \\ (\widehat{\boldsymbol{\theta}}_N^{(0)} - \boldsymbol{\theta}_N)^T\boldsymbol{Q}_{N,p}(\widetilde{\boldsymbol{\theta}}_N)\end{bmatrix}\Bigg\}(\widehat{\boldsymbol{\theta}}_N^{(0)} - \boldsymbol{\theta}_N).\end{aligned}$$

Multiplying through by $\{\boldsymbol{V}_N(\boldsymbol{\theta}_N)\}^{-1/2}$, we obtain

$$
\begin{aligned}
\{\boldsymbol{V}_N(\boldsymbol{\theta}_N)\}^{-1/2}(\widehat{\boldsymbol{\theta}}_N^{(1)} - \boldsymbol{\theta}_N) &= -\sqrt{N}\{\boldsymbol{M}_N(\boldsymbol{\theta}_N)\}^{-1/2} \overbrace{\boldsymbol{H}_N(\boldsymbol{\theta}_N)\left(\nabla_{\boldsymbol{\theta}}\boldsymbol{U}_N^{(N)}(\boldsymbol{\theta})\Big|_{\boldsymbol{\theta}=\widehat{\boldsymbol{\theta}}_N^{(0)}}\right)^{-1}}^{\xrightarrow{\mathbb{P}} I_p \text{ by (a)}} \boldsymbol{U}_N^{(N)}(\boldsymbol{\theta}_N) \\
&+ \sqrt{N}\{\boldsymbol{M}_N(\boldsymbol{\theta}_N)\}^{-1/2}\boldsymbol{H}_N(\boldsymbol{\theta}_N)\Bigg\{ \underbrace{I_p - \left(\nabla_{\boldsymbol{\theta}}\boldsymbol{U}_N^{(N)}(\boldsymbol{\theta})\Big|_{\boldsymbol{\theta}=\widehat{\boldsymbol{\theta}}_N^{(0)}}\right)^{-1} \nabla_{\boldsymbol{\theta}}\boldsymbol{U}_N^{(N)}(\boldsymbol{\theta})\Big|_{\boldsymbol{\theta}=\boldsymbol{\theta}_N}}_{\xrightarrow{\mathbb{P}} \mathbf{0}_{p\times p} \text{ by (b)}} \\
&- \underbrace{\left(\nabla_{\boldsymbol{\theta}}\boldsymbol{U}_N^{(N)}(\boldsymbol{\theta})\Big|_{\boldsymbol{\theta}=\widehat{\boldsymbol{\theta}}_N^{(0)}}\right)^{-1} \begin{bmatrix} (\widehat{\boldsymbol{\theta}}_N^{(0)} - \boldsymbol{\theta}_N)^T \boldsymbol{Q}_{N,1}(\widetilde{\boldsymbol{\theta}}_N) \\ \vdots \\ (\widehat{\boldsymbol{\theta}}_N^{(0)} - \boldsymbol{\theta}_N)^T \boldsymbol{Q}_{N,p}(\widetilde{\boldsymbol{\theta}}_N) \end{bmatrix}}_{= o_{\mathbb{P}}(1) \text{ by (c)}} \Bigg\}(\widehat{\boldsymbol{\theta}}_N^{(0)} - \boldsymbol{\theta}_N),
\end{aligned}
$$

where we invoked facts (a), (b), and (c) verified below. The first summand converges to a normal distribution by Lemma B.1 and the central limit theorem, whose application is justified under condition (ii). The second summand converges to zero in probability, as is seen by combining condition (vi) and another application of Lemma B.1—note that $\{\boldsymbol{M}_N(\boldsymbol{\theta}_N)\}^{1/2}$, $\boldsymbol{H}_N(\boldsymbol{\theta}_N)$, $\{\boldsymbol{M}_N(\boldsymbol{\theta}_N)\}^{-1/2}$, and $\{\boldsymbol{H}_N(\boldsymbol{\theta}_N)\}^{-1}$ are all $O_{\mathbb{P}}(1)$ under conditions (ii), (iii) and (iv).

It remains to verify the following facts:

(a) $\boldsymbol{H}_N(\boldsymbol{\theta}_N)\left(\nabla_{\boldsymbol{\theta}}\boldsymbol{U}_N^{(N)}(\boldsymbol{\theta})\Big|_{\boldsymbol{\theta}=\widehat{\boldsymbol{\theta}}_N^{(0)}}\right)^{-1} \xrightarrow{\mathbb{P}} I_p$

(b) $\left(\nabla_{\boldsymbol{\theta}}\boldsymbol{U}_N^{(N)}(\boldsymbol{\theta})\Big|_{\boldsymbol{\theta}=\widehat{\boldsymbol{\theta}}_N^{(0)}}\right)^{-1} \nabla_{\boldsymbol{\theta}}\boldsymbol{U}_N^{(N)}(\boldsymbol{\theta})\Big|_{\boldsymbol{\theta}=\boldsymbol{\theta}_N} \xrightarrow{\mathbb{P}} I_p$

(c) $\left(\nabla_{\boldsymbol{\theta}}\boldsymbol{U}_N^{(N)}(\boldsymbol{\theta})\Big|_{\boldsymbol{\theta}=\widehat{\boldsymbol{\theta}}_N^{(0)}}\right)^{-1} \begin{bmatrix} (\widehat{\boldsymbol{\theta}}_N^{(0)} - \boldsymbol{\theta}_N)^T \boldsymbol{Q}_{N,1}(\widetilde{\boldsymbol{\theta}}_N) \\ \vdots \\ (\widehat{\boldsymbol{\theta}}_N^{(0)} - \boldsymbol{\theta}_N)^T \boldsymbol{Q}_{N,p}(\widetilde{\boldsymbol{\theta}}_N) \end{bmatrix} \xrightarrow{\mathbb{P}} \mathbf{0}_{p\times p}$

Observe first that $\widehat{\boldsymbol{\theta}}_N^{(0)} - \boldsymbol{\theta}_N = o_{\mathbb{P}}(1)$ under our assumptions: this follows from condition (vi), and the fact that the matrices $\{\boldsymbol{M}_N(\boldsymbol{\theta}_N)\}^{1/2}$ and $\{\boldsymbol{H}_N(\boldsymbol{\theta}_N)\}^{-1}$ are bounded under conditions

(iii) and (iv). For fact (a), see that

$$\begin{aligned}
&\nabla_{\boldsymbol{\theta}} \boldsymbol{U}_N^{(N)}(\boldsymbol{\theta})\Big|_{\boldsymbol{\theta}=\widehat{\boldsymbol{\theta}}_N^{(0)}} - \boldsymbol{H}_N(\boldsymbol{\theta}_N) \\
&= \mathbb{P}_N\left[\nabla_{\boldsymbol{\theta}} \boldsymbol{U}_N(Z;\boldsymbol{\theta})|_{\boldsymbol{\theta}=\widehat{\boldsymbol{\theta}}_N^{(0)}}\right] - \mathbb{E}\left(\nabla_{\boldsymbol{\theta}} \boldsymbol{U}_N(Z;\boldsymbol{\theta})|_{\boldsymbol{\theta}=\boldsymbol{\theta}_N}\right) \\
&= \mathbb{P}_N\left[\nabla_{\boldsymbol{\theta}} \boldsymbol{U}_N(Z;\boldsymbol{\theta})|_{\boldsymbol{\theta}=\widehat{\boldsymbol{\theta}}_N^{(0)}} - \nabla_{\boldsymbol{\theta}} \boldsymbol{U}_N(Z;\boldsymbol{\theta})|_{\boldsymbol{\theta}=\boldsymbol{\theta}_N}\right] \\
&\qquad + \left\{\mathbb{P}_N\left[\nabla_{\boldsymbol{\theta}} \boldsymbol{U}_N(Z;\boldsymbol{\theta})|_{\boldsymbol{\theta}=\boldsymbol{\theta}_N}\right] - \mathbb{E}\left(\nabla_{\boldsymbol{\theta}} \boldsymbol{U}_N(Z;\boldsymbol{\theta})|_{\boldsymbol{\theta}=\boldsymbol{\theta}_N}\right)\right\}.
\end{aligned}$$

The first summand is bounded above by $C_1\|\widehat{\boldsymbol{\theta}}_N^{(0)} - \boldsymbol{\theta}_N\| = o_{\mathbb{P}}(1)$ by condition (i), and the second summand converges to zero by a weak law of large numbers, justified by condition (v). Thus, (a) holds by the continuous mapping theorem—note that all matrices involved are invertible and stochastically bounded under condition (iii). Fact (b) is shown using the same argument as for the first summand analyzed above for fact (a). Finally, fact (c) holds by condition (i) (i.e., the second derivative matrices are uniformly bounded), the fact that $\widehat{\boldsymbol{\theta}}_N^{(0)} - \boldsymbol{\theta}_N = o_{\mathbb{P}}(1)$, and condition (iii) (i.e., the left multiplying matrix is $O_{\mathbb{P}}(1)$), □

## B.1 Condition (vi)

To provide intuition for condition (vi) in the statement of Theorem B.2, we derive an interpretable set of conditions that imply condition (vi) in a special case of our general framework: a non-functional, univariate (i.e., $p = q = 1$), non-clustered (i.e., $n_i = 1\ \forall\ i \in [N]$) ridge regression that is weighted by the inverse of the working covariance matrix. This setting provides insight for the more general case: (1) the fully-iterated unweighted ridge is analogous to the initial penalized GEE estimator that is also unweighted (i.e., adopts an independence working covariance structure) and fully-iterated; (2) the fully-iterated weighted ridge is analogous to the fully-iterated and weighted (i.e., adopts some non-independence working covariance structure) penalized GEE. In this special case, we show that if the scaled smoothing parameters for the initial estimate satisfy $\frac{1}{N}\lambda_{0,N} = O(N^{-1/2})$, and for the weighted estimator satisfy $\frac{1}{N}\lambda_N = O(N^{-1/2})$, then condition (vi) holds. That is, if each of these scaled smoothing parameters individually (i.e., no conditions are required jointly on these rates) go to zero fast enough, then $\sqrt{N}\{\boldsymbol{M}_N(\boldsymbol{\theta}_N)\}^{-1/2}\boldsymbol{H}_N(\boldsymbol{\theta}_N)(\widehat{\boldsymbol{\theta}}_N^{(0)} - \boldsymbol{\theta}_N) = O_{\mathbb{P}}(1)$. Importantly, the smoothing parameter

rates we require are weaker than (i.e., implied by) the rates needed for the theoretical properties described in Chen et al. (2013). For example, even for the small knot setting, Chen et al. (2013) require that $\lambda = O(N^{\gamma})$ for $\gamma \leq (\tilde{p}+2-\tilde{q})/(2\tilde{p}+3)$. This implies, for instance, that for a $\tilde{p}^{th}$ order truncated polynomial using cubic B-splines, $\gamma \leq (\tilde{p}+2-(\tilde{p}+1))/(2\tilde{p}+3) = 1/9$. Put onto the scale of a single cluster, Chen et al. (2013) requires the faster rate of $\frac{1}{N}\lambda = O(N^{-8/9})$ than the $O(N^{-1/2})$ rate required by our theory in this special case. By this reasoning, condition (vi) is a weak assumption in this special case. Our conjecture is that this extends to more complicated settings although we omit such analysis in this work.

**Univariate Weighted Ridge** In the special case we explore, we define the following population parameters and estimators. At the outset, we define quantities with matrix notation to be consistent with the notation used in the remainder of the paper. Later on, we restrict our analysis to the $p = 1$ case for simplicity:

- Fully-Iterated (Population) Parameter (Closed-Form): for $\lambda_N$ such that $\frac{1}{N}\lambda_N \to 0$,

$$\begin{aligned}\beta_N &= \mathbb{E}(\widetilde{\mathrm{Var}}_N^{-1}(Y \mid X)[XX^T + \frac{1}{N}\lambda_N I_p])^{-1}\mathbb{E}(\widetilde{\mathrm{Var}}_N^{-1}(Y \mid X)[XY]) \\ &\overset{N\to\infty}{\to} \beta^* = \mathbb{E}(\mathrm{Var}^{-1}(Y \mid X)[XX^T])^{-1}\mathbb{E}(\mathrm{Var}^{-1}(Y \mid X)[XY]) \\ &\equiv [\mathbb{E}(XX^T)]^{-1}\mathbb{E}(XY)\end{aligned}$$

- Fully-Iterated Estimator (Closed-Form):

$$\widehat{\beta}_N^* = \mathbb{P}_N(\widetilde{\mathrm{Var}}_N^{-1}(Y \mid X)[XX^T + \frac{1}{N}\lambda_N I_p])^{-1}\mathbb{P}_N(\widetilde{\mathrm{Var}}_N^{-1}(Y \mid X)[XY])$$

- Unweighted Penalized (Initial Estimator) Population Parameter: if $\frac{1}{N}\lambda_{0,N} \to 0$,

$$\beta_N^{(0)} = \mathbb{E}(XX^T + \frac{1}{N}\lambda_{0,N} I_p)\mathbb{E}(XY) \overset{N\to\infty}{\to} \beta^*$$

- Unweighted Penalized (Initial) Estimator: $\widehat{\beta}_N^{(0)} = \mathbb{P}_N(XX^T + \frac{1}{N}\lambda_{0,N} I_p)\mathbb{P}_N(XY)$

- Coefficient Estimator Variance:

$$
\begin{aligned}
S_N(\beta) &= H_N^{-1}(\beta) M_N(\beta) H_N^{-1}(\beta) \\
&= \frac{1}{N}\{\mathbb{E}(\widetilde{\mathrm{Var}}_N^{-1}(Y \mid X)[XX^T + \frac{1}{N}\lambda_N I_p])\}^{-1} \Bigg[\mathbb{E}\left(\frac{\mathrm{Var}(Y \mid X)}{\widetilde{\mathrm{Var}}_N^{2}(Y \mid X)} XX^T\right) + \\
&\qquad \mathrm{Var}\{\widetilde{\mathrm{Var}}_N^{-1}(Y \mid X) XX^T(\beta - \beta^*)\}\Bigg] \{\mathbb{E}[\widetilde{\mathrm{Var}}_N^{-1}(Y \mid X) XX^T + \frac{1}{N}\lambda_N I_p]\}^{-1} \\
&\asymp \frac{1}{N}(\mathbb{E}[\mathrm{Var}^{-1}(Y \mid X) XX^T])^{-1} \quad \text{as } N \to \infty \text{ (if } \beta \to \beta^*)
\end{aligned}
$$

- Variance of Initial Coefficient Estimator

$$
\begin{aligned}
S_N^{(0)}(\beta) &= \frac{1}{N}(\mathbb{E}[XX^T + \frac{1}{N}\lambda_{0,N} I_p])^{-1}\left\{\mathbb{E}[\mathrm{Var}(Y \mid X) XX^T] + \mathrm{Var}[XX^T(\beta - \beta^*)]\right\}(\mathbb{E}[XX^T + \frac{1}{N}\lambda_{0,N} I_p \text{[illegible]} \\
&\asymp \frac{1}{N}(\mathbb{E}[XX^T])^{-1}\left\{\mathbb{E}[\mathrm{Var}(Y \mid X) XX^T]\right\}(\mathbb{E}[XX^T])^{-1} \quad \text{as } N \to \infty \text{ (if } \beta \to \beta^*)
\end{aligned}
$$

We now begin our exploration of this special case. First, observe that the above definitions imply, so long as $\frac{1}{N}\lambda_N \to 0$ and $\frac{1}{N}\lambda_{0,N} \to 0$,

$$
S_N^{-1}(\beta_N) S_N^{(0)}(\beta_N^{(0)}) \overset{N \to \infty}{\to} \mathbb{E}[\mathrm{Var}^{-1}(Y \mid X) XX^T](\mathbb{E}[XX^T])^{-1}\mathbb{E}[\mathrm{Var}(Y \mid X) XX^T](\mathbb{E}[XX^T])^{-1},
$$

so that $S_N^{-1}(\beta_N) S_N^{(0)}(\beta_N^{(0)}) = O(1)$.

Thus, when $p = 1$, the Taylor expansion for the quantity in condition (vi) has the form:

$$
\begin{aligned}
S_N^{-1/2}(\beta_N)\left(\widehat{\beta}_{0,N} - \beta_N\right) &\overset{p=1}{=} \frac{\widehat{\beta}_N^{(0)} - \beta_N^{(0)}}{\sqrt{S_N(\beta_N)}} + \frac{\beta_N^{(0)} - \beta_N}{\sqrt{S_N(\beta_N)}} \\
&= O(1) * \underbrace{\frac{\widehat{\beta}_N^{(0)} - \beta_N^{(0)}}{\sqrt{S_N^{(0)}(\beta_N^{(0)})}}}_{\substack{\overset{d}{\to} N(0,1) \\ \text{under weak} \\ \text{CLT conditions}}} \; + \; A
\end{aligned}
$$

where

$$A = \frac{\sqrt{N}\left\{\mathbb{E}\left(\frac{X^2}{\tilde{\sigma}^2(X)}\right) + \frac{1}{N}\lambda_N\right\}}{\sqrt{\mathbb{E}(\frac{\sigma^2(X)}{\tilde{\sigma}^4(X)}X^2) + (\beta_N - \beta^*)\mathrm{Var}(\frac{X^2}{\tilde{\sigma}^2(X)})}} * \frac{\frac{1}{N}\lambda_N \mathbb{E}(XY)\mathbb{E}(\frac{1}{\tilde{\sigma}^2(X)}) - \frac{1}{N}\lambda_{0,N}\mathbb{E}(XY/\tilde{\sigma}^2(X))}{\{\mathbb{E}(X^2) + \frac{1}{N}\lambda_{0,N}\}\{\mathbb{E}(\frac{X^2}{\tilde{\sigma}^2(X)}) + \frac{1}{N}\lambda_N \mathbb{E}(\frac{1}{\tilde{\sigma}^2(X)})}$$

$$= O(1) \quad \text{if } \frac{1}{N}\lambda_N = O(n^{-1/2}) \text{ and } \frac{1}{N}\lambda_{0,N} = O(n^{-1/2})$$

The above expansion shows that if, individually, $\frac{1}{N}\lambda_N = O(N^{-1/2})$ and $\frac{1}{N}\lambda_{0,N} = O(N^{-1/2})$, then the initial (unweighted) estimator converges at the same rate to $\beta_N$ as the fully-iterated (weighted) estimator. That is, in this special case, condition (vi) follows from the assumption that the scaled smoothing parameters of both estimators go to zero asymptotically at a rate of $1/\sqrt{n}$ or faster.

# Appendix C Additional Simulation Experiments

## C.1 Additional Simulations: Gaussian AR1

We simulated data from the model

$$Y_{i,j}(s) = \beta_0(s) + X_{1,i}\beta_1(s) + X_{2,i,j}\beta_2(s) + \epsilon_{i,j}(s) \tag{14}$$

where $\beta_0(s) = 3 + \sin(\pi s) + \sqrt{2}\cos(3\pi s)$, $\beta_1(s) = 3 + \cos(2\pi s) + \sqrt{2}\cos(3\pi s)$, $\beta_2(s) = 5\phi(\frac{s-0.35}{0.1}) - 5\phi(\frac{s-0.65}{0.2})$, $s \in \mathcal{S} \equiv [0,1]$, $|\mathcal{S}| = 100$, and $\phi(\cdot)$ denotes the standard normal density function. Basing our simulations off of those in Li et al. (2022), we drew $X_{1,i} \sim N(0,1)$, and $X_{2,i,j} = j + e_{i,j}$, where $e_{i,j} \sim N(\alpha e_{i,j-1}, 1)$, with $e_{i,0} = 0$, $\alpha = 0.7$.

We first assess performance in simulations where the marginal covariance of the functional outcome has the structure adopted in our estimator: the $Sn_i \times Sn_i$ covariance matrix $\mathrm{Cov}(\boldsymbol{Y}_i \mid \mathbb{X}_i) = \mathrm{Cov}(\boldsymbol{\epsilon}_i) = \mathrm{blockdiag}(\Sigma_i(1), \ldots, \Sigma_i(S))$, where $\Sigma(s) = \mathrm{Cov}(\boldsymbol{Y}_i(s) \mid \mathbb{X}_i) = \mathrm{Cov}(\boldsymbol{\epsilon}_i(s)) \in \mathbb{R}^{n_i \times n_i}$. We simulated data with the pointwise AR1 correlation structure $\Sigma_{i,j}(s) = \sigma_\epsilon^2 \rho^{|i-j|}$ for all $s \in \mathcal{S}$. We set $\sigma_\epsilon^2 = 10$ and $\rho \in \{0.25, 0.5, 0.75\}$.

We compared the One-Step to three benchmarks: 1) a penalized GLS with an independence working correlation structure (GLS-Ind), 2) a GLS with an exchangeable correlation structure

(GLS-Ex), and 3) the initial FoSR estimator, $\widehat{\boldsymbol{\beta}}^{(0)}_{\Lambda_0}$ (fit with `pffr`). We constructed CIs using a sandwich estimator (Chen et al., 2013) for all methods, using the corresponding independence or exchangeable $\mathbb{V}_i$ forms. We show in Appendix Table 20 that coverage is comparable between CIs constructed with sandwich and fast bootstrap variance estimators. Benchmark 1) shows how our implementation and tuning scheme performs without exploiting intra-cluster correlation, 2) shows performance of an estimator similar to a fully-iterated version of the One-Step fGEE (using the same exchangeable correlation structure), and 3) shows the performance of a FoSR that ignores intra-cluster correlation. CIs for the FoSR fit (benchmark 3) should, however, achieve nominal coverage in this correlated setting, given that we use a sandwich variance estimator.

Table 12 shows that the One-Step improves estimation performance compared to $\widehat{\boldsymbol{\theta}}^{(0)}_{\Lambda_0}$ (FoSR) and the GLS-Ind. One-Step performance is comparable to that of the GLS-AR1, suggesting that it is asymptotically as efficient as the fully iterated GEE. Table 13 shows that the FoSR yields overly conservative pointwise and joint CIs, while the One-Step yields roughly nominal coverage, although inference is slightly anti-conservative when both $N$ and $n_i$ grow large. Coverage of the FoSR appears to also drop with large $N$ and $n_i$. Table 14 shows that the FoSR yields overly conservative pointwise CIs, while the One-Step and GLS methods are a bit anti-conservative. All methods grow more anti-conservative as $n_i$ grows. Table 15 shows that the One-Step is fast, taking only 2.5-3 minutes to fit on a dataset with $N = 100$, and $n_i = 100$, without parallelization.

Together these results suggest that 1) the One-Step improves estimation accuracy compared to working independence methods, 2) is as efficient as the fully iterated GEE (given similarity in performance to the GLS) in even moderate sample sizes, 3) is scalable to large cluster sizes and numbers, and 4) achieves approximately nominal coverage.

## C.2 Gaussian Exchangeable

Here we simulate data as in main text Section 4.2 "Simulation 2: Gaussian Outcome with Exchangeable Correlation," except that we set $\xi_{i,1} \overset{\text{iid}}{\sim} N(0,5)$, $\xi_{i,2} \overset{\text{iid}}{\sim} N(0,2)$, $\zeta_{i,j,1} \overset{\text{iid}}{\sim} N(0,3)$ and $\xi_{i,j,2} \overset{\text{iid}}{\sim} N(0,1)$. $\epsilon_{i,j}(s) \overset{\text{iid}}{\sim} N(0,10)$. We set $\sigma^2_\epsilon = 10$.

| $N$ | $n_i$ | One-Step | | | GLS-AR1 | | | GLS-Ind | | |
|---|---|---|---|---|---|---|---|---|---|---|
| | | 0.25 | 0.5 | 0.75 | 0.25 | 0.5 | 0.75 | 0.25 | 0.5 | 0.75 |
| 25 | 5 | 0.99 ± 0.00 | 0.97 ± 0.00 | 0.93 ± 0.00 | 1.01 ± 0.00 | 0.98 ± 0.00 | 0.95 ± 0.00 | 1.00 ± 0.00 | 0.97 ± 0.00 | 0.94 ± 0.00 |
| | 25 | 0.96 ± 0.00 | 0.92 ± 0.00 | 0.85 ± 0.00 | 0.98 ± 0.00 | 0.93 ± 0.00 | 0.86 ± 0.00 | 0.98 ± 0.00 | 0.95 ± 0.00 | 0.91 ± 0.00 |
| | 100 | 0.97 ± 0.00 | 0.93 ± 0.00 | 0.87 ± 0.00 | 0.99 ± 0.00 | 0.95 ± 0.00 | 0.88 ± 0.00 | 1.00 ± 0.00 | 0.97 ± 0.00 | 0.93 ± 0.00 |
| 50 | 5 | 0.98 ± 0.00 | 0.96 ± 0.00 | 0.93 ± 0.00 | 0.99 ± 0.00 | 0.97 ± 0.00 | 0.93 ± 0.00 | 0.98 ± 0.00 | 0.96 ± 0.00 | 0.93 ± 0.00 |
| | 25 | 0.97 ± 0.00 | 0.93 ± 0.00 | 0.86 ± 0.00 | 0.97 ± 0.00 | 0.93 ± 0.00 | 0.86 ± 0.00 | 0.98 ± 0.00 | 0.95 ± 0.00 | 0.91 ± 0.00 |
| | 100 | 0.97 ± 0.00 | 0.94 ± 0.00 | 0.88 ± 0.00 | 0.98 ± 0.00 | 0.95 ± 0.00 | 0.89 ± 0.00 | 0.98 ± 0.00 | 0.97 ± 0.00 | 0.93 ± 0.00 |
| 100 | 5 | 0.97 ± 0.00 | 0.95 ± 0.00 | 0.93 ± 0.00 | 0.98 ± 0.00 | 0.96 ± 0.00 | 0.93 ± 0.00 | 0.97 ± 0.00 | 0.95 ± 0.00 | 0.93 ± 0.00 |
| | 25 | 0.96 ± 0.00 | 0.92 ± 0.00 | 0.86 ± 0.00 | 0.97 ± 0.00 | 0.93 ± 0.00 | 0.86 ± 0.00 | 0.97 ± 0.00 | 0.95 ± 0.00 | 0.91 ± 0.00 |
| | 100 | 0.97 ± 0.00 | 0.95 ± 0.00 | 0.90 ± 0.00 | 0.98 ± 0.00 | 0.95 ± 0.00 | 0.90 ± 0.00 | 0.99 ± 0.00 | 0.97 ± 0.00 | 0.94 ± 0.00 |

Table 12: Functional Coefficient Estimation Performance relative to initial FoSR fit, which assumes working independence correlation structure ($\text{RMSE}_{AR1}/\text{RMSE}_{FoSR}$). Cells contain the average of 300 replicates ± SEM (SEM= 0.00 indicates a value < 0.01).

| $N$ | $n_i$ | One-Step | | | GLS-AR1 | | | GLS-Ind | | | FoSR | | |
|---|---|---|---|---|---|---|---|---|---|---|---|---|---|
| | | 0.25 | 0.5 | 0.75 | 0.25 | 0.5 | 0.75 | 0.25 | 0.5 | 0.75 | 0.25 | 0.5 | 0.75 |
| 25 | 5 | 0.97 ± 0.00 | 0.97 ± 0.00 | 0.97 ± 0.00 | 0.97 ± 0.00 | 0.97 ± 0.00 | 0.97 ± 0.00 | 0.97 ± 0.00 | 0.97 ± 0.00 | 0.97 ± 0.00 | 1.00 ± 0.00 | 1.00 ± 0.00 | 1.00 ± 0.00 |
| | 25 | 0.97 ± 0.00 | 0.97 ± 0.00 | 0.97 ± 0.00 | 0.97 ± 0.00 | 0.97 ± 0.00 | 0.97 ± 0.00 | 0.97 ± 0.00 | 0.97 ± 0.00 | 0.97 ± 0.00 | 1.00 ± 0.00 | 1.00 ± 0.00 | 1.00 ± 0.00 |
| | 100 | 0.95 ± 0.00 | 0.95 ± 0.00 | 0.94 ± 0.00 | 0.95 ± 0.00 | 0.95 ± 0.00 | 0.94 ± 0.00 | 0.95 ± 0.00 | 0.96 ± 0.00 | 0.97 ± 0.00 | 0.98 ± 0.00 | 0.99 ± 0.00 | 0.99 ± 0.00 |
| 50 | 5 | 0.98 ± 0.00 | 0.98 ± 0.00 | 0.98 ± 0.00 | 0.98 ± 0.00 | 0.98 ± 0.00 | 0.98 ± 0.00 | 0.98 ± 0.00 | 0.98 ± 0.00 | 0.98 ± 0.00 | 1.00 ± 0.00 | 1.00 ± 0.00 | 1.00 ± 0.00 |
| | 25 | 0.97 ± 0.00 | 0.97 ± 0.00 | 0.97 ± 0.00 | 0.97 ± 0.00 | 0.97 ± 0.00 | 0.97 ± 0.00 | 0.97 ± 0.00 | 0.98 ± 0.00 | 0.98 ± 0.00 | 1.00 ± 0.00 | 1.00 ± 0.00 | 1.00 ± 0.00 |
| | 100 | 0.94 ± 0.00 | 0.94 ± 0.00 | 0.92 ± 0.00 | 0.94 ± 0.00 | 0.94 ± 0.00 | 0.92 ± 0.00 | 0.94 ± 0.00 | 0.96 ± 0.00 | 0.97 ± 0.00 | 0.97 ± 0.00 | 0.98 ± 0.00 | 0.98 ± 0.00 |
| 100 | 5 | 0.98 ± 0.00 | 0.99 ± 0.00 | 0.99 ± 0.00 | 0.99 ± 0.00 | 0.99 ± 0.00 | 0.99 ± 0.00 | 0.99 ± 0.00 | 0.99 ± 0.00 | 0.99 ± 0.00 | 1.00 ± 0.00 | 1.00 ± 0.00 | 1.00 ± 0.00 |
| | 25 | 0.96 ± 0.00 | 0.96 ± 0.00 | 0.96 ± 0.00 | 0.96 ± 0.00 | 0.97 ± 0.00 | 0.96 ± 0.00 | 0.97 ± 0.00 | 0.97 ± 0.00 | 0.98 ± 0.00 | 1.00 ± 0.00 | 1.00 ± 0.00 | 1.00 ± 0.00 |
| | 100 | 0.91 ± 0.00 | 0.92 ± 0.00 | 0.90 ± 0.00 | 0.91 ± 0.00 | 0.92 ± 0.00 | 0.90 ± 0.00 | 0.92 ± 0.00 | 0.94 ± 0.00 | 0.95 ± 0.00 | 0.93 ± 0.00 | 0.96 ± 0.00 | 0.97 ± 0.00 |

Table 13: Functional Coefficient Joint 95% Confidence Interval Coverage. Data are simulated with an AR1 correlation structure. Cells contain the average of 300 replicates ± SEM (SEM= 0.00 indicates a value < 0.01).

| $N$ | $n_i$ | One-Step | | | GLS-AR1 | | | GLS-Ind | | | FoSR | | |
|---|---|---|---|---|---|---|---|---|---|---|---|---|---|
| | | 0.25 | 0.5 | 0.75 | 0.25 | 0.5 | 0.75 | 0.25 | 0.5 | 0.75 | 0.25 | 0.5 | 0.75 |
| 25 | 5 | 0.89 ± 0.00 | 0.89 ± 0.00 | 0.89 ± 0.00 | 0.90 ± 0.00 | 0.89 ± 0.00 | 0.89 ± 0.00 | 0.90 ± 0.00 | 0.89 ± 0.00 | 0.89 ± 0.00 | 0.99 ± 0.00 | 0.99 ± 0.00 | 0.99 ± 0.00 |
| | 25 | 0.89 ± 0.00 | 0.89 ± 0.00 | 0.88 ± 0.00 | 0.89 ± 0.00 | 0.89 ± 0.00 | 0.88 ± 0.00 | 0.89 ± 0.00 | 0.89 ± 0.00 | 0.89 ± 0.00 | 0.99 ± 0.00 | 0.99 ± 0.00 | 0.99 ± 0.00 |
| | 100 | 0.87 ± 0.00 | 0.87 ± 0.00 | 0.85 ± 0.00 | 0.86 ± 0.00 | 0.87 ± 0.00 | 0.85 ± 0.00 | 0.87 ± 0.00 | 0.88 ± 0.00 | 0.89 ± 0.00 | 0.96 ± 0.00 | 0.96 ± 0.00 | 0.95 ± 0.00 |
| 50 | 5 | 0.91 ± 0.00 | 0.91 ± 0.00 | 0.91 ± 0.00 | 0.91 ± 0.00 | 0.91 ± 0.00 | 0.91 ± 0.00 | 0.91 ± 0.00 | 0.91 ± 0.00 | 0.91 ± 0.00 | 0.99 ± 0.00 | 0.99 ± 0.00 | 0.99 ± 0.00 |
| | 25 | 0.90 ± 0.00 | 0.90 ± 0.00 | 0.89 ± 0.00 | 0.90 ± 0.00 | 0.90 ± 0.00 | 0.89 ± 0.00 | 0.90 ± 0.00 | 0.90 ± 0.00 | 0.91 ± 0.00 | 0.99 ± 0.00 | 0.99 ± 0.00 | 0.99 ± 0.00 |
| | 100 | 0.86 ± 0.00 | 0.86 ± 0.00 | 0.84 ± 0.00 | 0.86 ± 0.00 | 0.86 ± 0.00 | 0.84 ± 0.00 | 0.86 ± 0.00 | 0.88 ± 0.00 | 0.89 ± 0.00 | 0.93 ± 0.00 | 0.94 ± 0.00 | 0.95 ± 0.00 |
| 100 | 5 | 0.91 ± 0.00 | 0.92 ± 0.00 | 0.92 ± 0.00 | 0.92 ± 0.00 | 0.92 ± 0.00 | 0.92 ± 0.00 | 0.92 ± 0.00 | 0.92 ± 0.00 | 0.92 ± 0.00 | 0.99 ± 0.00 | 0.99 ± 0.00 | 0.99 ± 0.00 |
| | 25 | 0.89 ± 0.00 | 0.89 ± 0.00 | 0.88 ± 0.00 | 0.89 ± 0.00 | 0.89 ± 0.00 | 0.88 ± 0.00 | 0.89 ± 0.00 | 0.90 ± 0.00 | 0.91 ± 0.00 | 0.99 ± 0.00 | 0.99 ± 0.00 | 0.99 ± 0.00 |
| | 100 | 0.83 ± 0.00 | 0.84 ± 0.00 | 0.82 ± 0.00 | 0.84 ± 0.00 | 0.85 ± 0.00 | 0.83 ± 0.00 | 0.84 ± 0.00 | 0.86 ± 0.00 | 0.87 ± 0.00 | 0.89 ± 0.00 | 0.91 ± 0.00 | 0.94 ± 0.00 |

Table 14: Functional Coefficient Pointwise 95% Confidence Interval Coverage. Data are simulated with an AR1 correlation structure. Cells contain the average of 300 replicates ± SEM (SEM= 0.00 indicates a value < 0.01).

Table 16 shows the estimation performance and illustrates that the One-Step performs comparably to the GLS-Ex, suggesting the One-Step performs comparably to a fully-iterated GEE. Moreover the One-Step performs comparably to the marginal approach (Li et al., 2022) that models correlation across the functional domain (both within- and across longitudinal observations of the functional outcome). Thus, in these simulations, modeling correlation at each point $s$, is enough to capture efficiency gains. Table 18 shows that the pointwise coverage of the One-Step is often close to the nominal levels. In contrast, the Marginal approach is often highly anti-conservative for large $n_i$, a quality acknowledged in (Li et al., 2022). The GLS approaches

| $N$ | $n_i$ | One-Step | | | GLS-AR1 | | | GLS-Ind | | | FoSR | | |
|---|---|---|---|---|---|---|---|---|---|---|---|---|---|
| | | 0.25 | 0.5 | 0.75 | 0.25 | 0.5 | 0.75 | 0.25 | 0.5 | 0.75 | 0.25 | 0.5 | 0.75 |
| 25 | 5 | 16.54 ± 0.10 | 16.21 ± 0.07 | 24.79 ± 0.18 | 8.35 ± 0.04 | 8.20 ± 0.02 | 12.08 ± 0.01 | 9.69 ± 0.05 | 9.55 ± 0.04 | 12.26 ± 0.02 | 0.15 ± 0.00 | 0.15 ± 0.00 | 0.22 ± 0.00 |
| | 25 | 22.56 ± 0.15 | 20.80 ± 0.12 | 20.47 ± 0.11 | 12.28 ± 0.10 | 11.55 ± 0.08 | 11.13 ± 0.05 | 12.52 ± 0.11 | 11.78 ± 0.09 | 11.23 ± 0.07 | 0.28 ± 0.00 | 0.27 ± 0.00 | 0.26 ± 0.00 |
| | 100 | 45.85 ± 0.58 | 39.32 ± 0.17 | 41.68 ± 0.50 | 34.17 ± 0.50 | 29.34 ± 0.31 | 28.54 ± 0.27 | 30.27 ± 0.46 | 26.13 ± 0.22 | 26.24 ± 0.27 | 1.05 ± 0.02 | 0.72 ± 0.00 | 0.73 ± 0.01 |
| 50 | 5 | 24.18 ± 0.15 | 22.93 ± 0.14 | 23.26 ± 0.06 | 14.60 ± 0.09 | 14.44 ± 0.08 | 14.16 ± 0.03 | 16.29 ± 0.09 | 16.22 ± 0.10 | 16.21 ± 0.08 | 0.22 ± 0.00 | 0.21 ± 0.00 | 0.21 ± 0.00 |
| | 25 | 33.37 ± 0.22 | 32.74 ± 0.28 | 33.42 ± 0.25 | 21.95 ± 0.24 | 21.54 ± 0.15 | 22.68 ± 0.25 | 21.62 ± 0.19 | 21.32 ± 0.13 | 22.30 ± 0.20 | 0.48 ± 0.00 | 0.48 ± 0.00 | 0.49 ± 0.00 |
| | 100 | 82.80 ± 1.03 | 84.35 ± 1.11 | 86.85 ± 1.19 | 61.59 ± 0.72 | 69.19 ± 1.03 | 68.34 ± 1.02 | 53.39 ± 0.65 | 60.84 ± 0.92 | 59.75 ± 0.90 | 1.54 ± 0.02 | 1.79 ± 0.03 | 1.66 ± 0.03 |
| 100 | 5 | 37.37 ± 0.13 | 36.62 ± 0.12 | 37.55 ± 0.13 | 26.49 ± 0.09 | 26.45 ± 0.08 | 26.23 ± 0.07 | 27.42 ± 0.11 | 28.07 ± 0.16 | 27.67 ± 0.13 | 0.30 ± 0.00 | 0.31 ± 0.00 | 0.30 ± 0.00 |
| | 25 | 62.10 ± 0.80 | 70.89 ± 0.20 | 57.45 ± 0.47 | 46.39 ± 0.48 | 59.69 ± 0.61 | 46.66 ± 0.57 | 42.84 ± 0.30 | 52.79 ± 0.25 | 41.42 ± 0.40 | 0.77 ± 0.00 | 1.24 ± 0.00 | 0.76 ± 0.00 |
| | 100 | 187.81 ± 3.17 | 175.73 ± 2.32 | 176.43 ± 2.67 | 159.26 ± 2.62 | 161.64 ± 2.81 | 144.59 ± 2.33 | 131.43 ± 5.45 | 122.23 ± 1.69 | 114.57 ± 1.50 | 3.17 ± 0.08 | 4.16 ± 0.09 | 3.09 ± 0.04 |

Table 15: Fit Time (seconds) for the entire One-Step estimation procedure including 1) estimating the initial $\widehat{\boldsymbol{\beta}}^{(0)}_{\Lambda_0}$, 2) estimating correlation parameters, 3) tuning $\Lambda_1$, 4) calculating the One-Step $\widehat{\boldsymbol{\beta}}^{(1)}_{\Lambda_1}$, and 5) estimating $\widehat{\text{Var}}(\widehat{\boldsymbol{\beta}}^{(1)}_{\Lambda_1})$ and constructing CIs. Data are simulated with an AR1 correlation structure. Cells contain the average of 300 replicates ± SEM (SEM= 0.00 indicates a value $< 0.01$).

| $N$ | $n_i$ | One-Step | GLS-Ex | GLS-Ind | Marginal |
|---|---|---|---|---|---|
| 25 | 5 | 0.96 ± 0.00 | 0.96 ± 0.00 | 1.00 ± 0.00 | 0.96 ± 0.01 |
| | 25 | 0.97 ± 0.00 | 0.97 ± 0.00 | 1.00 ± 0.00 | 0.99 ± 0.01 |
| | 100 | 1.00 ± 0.00 | 1.00 ± 0.00 | 1.00 ± 0.00 | 1.01 ± 0.01 |
| 50 | 5 | 0.95 ± 0.01 | 0.95 ± 0.01 | 0.99 ± 0.00 | 0.95 ± 0.01 |
| | 25 | 0.97 ± 0.00 | 0.97 ± 0.00 | 1.00 ± 0.00 | 0.98 ± 0.01 |
| | 100 | 0.99 ± 0.00 | 0.99 ± 0.00 | 1.00 ± 0.00 | 1.02 ± 0.01 |
| 100 | 5 | 0.95 ± 0.01 | 0.95 ± 0.00 | 0.99 ± 0.00 | 0.94 ± 0.01 |
| | 25 | 0.97 ± 0.00 | 0.97 ± 0.00 | 1.00 ± 0.00 | 0.97 ± 0.01 |
| | 100 | 0.99 ± 0.00 | 0.99 ± 0.00 | 1.00 ± 0.00 | — |

Table 16: Gaussian Exchangeable: RMSE relative to the relative to the initial FoSR fit $\widehat{\boldsymbol{\theta}}^{(0)}_{\Lambda_0}$ (fit with `pffr()`). We indicate out-of-memory (30Gb) with the symbol — . Cells contain the average of 300 replicates ± SEM (SEM= 0.00 indicates a value $< 0.01$).

are often highly conservative, while the FoSR is slightly more anti-conservative compared to the One-Step in smaller samples. Table 17 shows that the joint coverage tends to be slightly more conservative than the FoSR, but both are substantially less conservative than the GLS approaches.

| $N$ | $n_i$ | One-Step | GLS-Ex | GLS-Ind | FoSR |
|---|---|---|---|---|---|
| 25 | 5 | 0.97 ± 0.00 | 1.00 ± 0.00 | 1.00 ± 0.00 | 0.95 ± 0.01 |
| | 25 | 0.97 ± 0.00 | 1.00 ± 0.00 | 1.00 ± 0.00 | 0.97 ± 0.00 |
| | 100 | 0.98 ± 0.00 | 1.00 ± 0.00 | 1.00 ± 0.00 | 0.97 ± 0.00 |
| 50 | 5 | 0.99 ± 0.00 | 1.00 ± 0.00 | 1.00 ± 0.00 | 0.95 ± 0.01 |
| | 25 | 0.99 ± 0.00 | 1.00 ± 0.00 | 1.00 ± 0.00 | 0.98 ± 0.00 |
| | 100 | 0.98 ± 0.00 | 1.00 ± 0.00 | 1.00 ± 0.00 | 0.98 ± 0.00 |
| 100 | 5 | 0.99 ± 0.00 | 1.00 ± 0.00 | 1.00 ± 0.00 | 0.96 ± 0.00 |
| | 25 | 0.99 ± 0.00 | 1.00 ± 0.00 | 1.00 ± 0.00 | 0.99 ± 0.00 |
| | 100 | 0.99 ± 0.00 | 1.00 ± 0.00 | 1.00 ± 0.00 | 0.98 ± 0.00 |

Table 17: Gaussian Exchangeable: Joint 95% CI coverage. Cells contain the average of 300 replicates ± SEM (SEM= 0.00 indicates a value $< 0.01$).

| $N$ | $n_i$ | One-Step | GLS-Ex | GLS-Ind | Marginal | FoSR |
|---|---|---|---|---|---|---|
| 25 | 5 | 0.92 ± 0.00 | 0.96 ± 0.00 | 0.96 ± 0.00 | 0.94 ± 0.00 | 0.90 ± 0.00 |
| | 25 | 0.92 ± 0.00 | 0.95 ± 0.00 | 0.96 ± 0.00 | 0.94 ± 0.00 | 0.92 ± 0.00 |
| | 100 | 0.94 ± 0.00 | 0.97 ± 0.00 | 0.96 ± 0.00 | 0.71 ± 0.00 | 0.93 ± 0.00 |
| 50 | 5 | 0.95 ± 0.00 | 0.97 ± 0.00 | 0.97 ± 0.00 | 0.94 ± 0.00 | 0.91 ± 0.00 |
| | 25 | 0.95 ± 0.00 | 0.97 ± 0.00 | 0.97 ± 0.00 | 0.95 ± 0.00 | 0.94 ± 0.00 |
| | 100 | 0.94 ± 0.00 | 0.96 ± 0.00 | 0.97 ± 0.00 | 0.70 ± 0.00 | 0.93 ± 0.00 |
| 100 | 5 | 0.96 ± 0.00 | 0.98 ± 0.00 | 0.97 ± 0.00 | 0.95 ± 0.00 | 0.92 ± 0.00 |
| | 25 | 0.96 ± 0.00 | 0.98 ± 0.00 | 0.98 ± 0.00 | 0.95 ± 0.00 | 0.94 ± 0.00 |
| | 100 | 0.96 ± 0.00 | 0.97 ± 0.00 | 0.98 ± 0.00 | — | 0.95 ± 0.00 |

Table 18: Gaussian Exchangeable: Pointwise CI coverage. We indicate out-of-memory (30Gb) with the symbol — . Cells contain the average of 300 replicates ± SEM (SEM= 0.00 indicates a value $< 0.01$).

| $N$ | $n_i$ | One-Step | GLS-Ex | GLS-Ind | Marginal | FoSR |
|---|---|---|---|---|---|---|
| 25 | 5 | 20.66 ± 0.14 | 9.05 ± 0.07 | 8.05 ± 0.04 | 0.49 ± 0.00 | 0.16 ± 0.00 |
| | 25 | 23.24 ± 0.10 | 11.98 ± 0.04 | 11.99 ± 0.05 | 4.85 ± 0.03 | 0.27 ± 0.00 |
| | 100 | 54.87 ± 0.59 | 35.85 ± 0.55 | 35.03 ± 0.54 | 75.15 ± 1.07 | 1.17 ± 0.01 |
| 50 | 5 | 27.45 ± 0.18 | 16.00 ± 0.13 | 14.60 ± 0.11 | 1.15 ± 0.01 | 0.21 ± 0.00 |
| | 25 | 34.59 ± 0.14 | 21.37 ± 0.10 | 19.97 ± 0.13 | 29.79 ± 0.27 | 0.45 ± 0.00 |
| | 100 | 86.76 ± 1.05 | 64.70 ± 0.51 | 66.35 ± 0.63 | 415.64 ± 2.57 | 2.15 ± 0.01 |
| 100 | 5 | 39.33 ± 0.21 | 27.26 ± 0.13 | 26.51 ± 0.14 | 3.79 ± 0.03 | 0.31 ± 0.00 |
| | 25 | 73.25 ± 0.90 | 54.20 ± 0.73 | 51.83 ± 0.74 | 233.72 ± 1.69 | 1.08 ± 0.02 |
| | 100 | 146.26 ± 2.27 | 102.88 ± 1.43 | 87.89 ± 0.99 | — | 2.79 ± 0.01 |

Table 19: Gaussian Exchangeable: Time to fit. We indicate out-of-memory (30Gb) with the symbol — . Cells contain the average of 300 replicates ± SEM (SEM= 0.00 indicates a value $< 0.01$).

## C.3 Comparison of Variance Estimators

Here we compare the performance of different strategies to estimate coefficient estimator variance and construct pointwise confidence intervals. The results below are from the simulation experiments described in main text Section 4.1, "Simulation 2: Gaussian Outcome with Exchangeable Correlation."

Table 20 shows that pointwise CIs constructed using $\widehat{\text{Var}}\left(\widehat{\boldsymbol{\theta}}_{\Lambda_1}^{(1)}\right)$, estimated with the sandwich or fast cluster bootstrap, achieve comparable coverage. This suggests that the fast cluster bootstrap accurately estimates $\widehat{\text{Var}}\left(\widehat{\boldsymbol{\theta}}_{\Lambda_1}^{(1)}\right)$ and can be used instead of the sandwich estimator.

Table 21 shows that the sandwich and fast cluster bootstrap strategies take comparable time as both require inversion of one $p \times p$ matrix. Taken together the sandwich estimator and fast cluster bootstrap yield comparable estimation accuracy and take comparable amounts of time.

| $N$ | $n_i$ | Fast Bootstrap | Sandwich |
|---|---|---|---|
| 25 | 5 | 0.92 ± 0.01 | 0.93 ± 0.01 |
| | 25 | 0.92 ± 0.01 | 0.93 ± 0.01 |
| | 100 | 0.94 ± 0.01 | 0.95 ± 0.00 |
| 50 | 5 | 0.94 ± 0.01 | 0.95 ± 0.00 |
| | 25 | 0.95 ± 0.00 | 0.95 ± 0.00 |
| | 100 | 0.94 ± 0.01 | 0.94 ± 0.01 |
| 100 | 5 | 0.95 ± 0.00 | 0.96 ± 0.00 |
| | 25 | 0.96 ± 0.00 | 0.96 ± 0.00 |
| | 100 | 0.95 ± 0.00 | 0.96 ± 0.00 |

Table 20: Pointwise CIs, constructed using $\widehat{\text{Var}}\left(\widehat{\boldsymbol{\theta}}_{\Lambda_1}^{(1)}\right)$ estimated with the sandwich or the fast cluster bootstrap, achieve comparable coverage. These results are from the Gaussian Exchangeable data simulations from main text Section 4.1. This shows that the fast cluster bootstrap performs well as an estimator for $\text{Var}\left(\widehat{\boldsymbol{\theta}}_{\Lambda_1}^{(1)}\right)$ and can be used as an alternative to the sandwich estimator. Cells contain the average of 300 replicates ± SEM (SEM= 0.00 indicates a value $< 0.01$).

| $N$ | $n_i$ | Fast Bootstrap | Sandwich |
|---|---|---|---|
| 25 | 5 | $19.63 \pm 0.20$ | $17.66 \pm 0.08$ |
| | 25 | $25.86 \pm 0.23$ | $24.63 \pm 0.15$ |
| | 100 | $44.96 \pm 0.19$ | $40.60 \pm 0.42$ |
| 50 | 5 | $25.69 \pm 0.10$ | $25.77 \pm 0.10$ |
| | 25 | $39.01 \pm 0.33$ | $34.75 \pm 0.30$ |
| | 100 | $77.62 \pm 0.45$ | $82.05 \pm 1.04$ |
| 100 | 5 | $37.52 \pm 0.14$ | $37.78 \pm 0.14$ |
| | 25 | $57.95 \pm 0.34$ | $58.95 \pm 0.46$ |
| | 100 | $147.34 \pm 1.32$ | $151.24 \pm 3.15$ |

Table 21: Total time of fitting the One-Step (sec), including tuning $\Lambda_1$, and estimating $\text{Var}\left(\widehat{\boldsymbol{\theta}}^{(1)}_{\Lambda_1}\right)$, when using sandwich and fast cluster bootstrap variance estimators. The differences in the timing between columns are thus entirely determined by variance estimators, $\text{Var}\left(\widehat{\boldsymbol{\theta}}^{(1)}_{\Lambda_1}\right)$. The relative speed of the sandwich and the fast cluster bootstrap depend on $N$ and $n_i$, but are mostly comparable. This shows that the fast cluster bootstrap can be used as an alternative to the sandwich estimator. Cells contain the average of 300 replicates ± SEM.

## C.4 Comparison of Joint CIs constructed with Parametric and Non-Parametric Bootstrap Based Strategies

Here we present the coverage of joint CIs constructed with a non-parametric bootstrap based strategy. This approaches is based on Section "BUILDING SIMULTANEOUS CONFIDENCE BANDS" in (Degras, 2017). However, we estimate the empirical quantile used to construct CIs in terms of resampling $\widehat{\boldsymbol{\theta}}^{(1)}$ instead of $\widehat{\boldsymbol{\beta}}^{(1)}$. The joint CI coverage of the non-parametric cluster bootstrap-based approach is similar to the coverage achieved by the parametric bootstrap-based strategy for joint CI construction, presented in main text 3. This shows that the the non-parametric cluster bootstrap-based approach can also be used to construct joint CIs. We emphasize that the non-parametric bootstrap strategy explored here calculates $\tilde{q}^{(r)}_{1-\alpha}$ using a non-parametric bootstrap. It is distinct from an approach that simply estimates $\text{Var}\left(\widehat{\boldsymbol{\theta}}^{(1)}_{\Lambda_1}\right)$ with a non-parametric cluster bootstrap and then calculates the empirical quantile of the joint CI, $\tilde{q}^{(r)}_{1-\alpha}$, using a parametric bootstrap.

| $N$ | $n_i$ | Non-Parametric |
|---|---|---|
| 25 | 5 | 0.97 ± 0.00 |
| | 25 | 0.97 ± 0.00 |
| | 100 | 0.98 ± 0.00 |
| 50 | 5 | 0.99 ± 0.00 |
| | 25 | 0.99 ± 0.00 |
| | 100 | 0.98 ± 0.00 |
| 100 | 5 | 0.99 ± 0.00 |
| | 25 | 0.99 ± 0.00 |

Table 22: Joint 95% CI Coverage for joint CIs constructed with a non-parametric cluster bootstrap-based approach. These results are from the Gaussian Exchangeable data simulations from main text Section 4.1. Cells contain the average of 300 replicates ± SEM (SEM= 0.00 indicates a value $< 0.01$).

# Appendix D Additional Application Analyses

## D.1 Whisker Activity

Since the authors were interested in how cells from this brain region encoded the sensory input of whisker activity, they recorded how much the whiskers moved when they were experimentally manipulated. The movement-by-moment activity of the whiskers can thus be used as a functional covariate to estimate how the association evolves across time. We fit the following concurrent model

$$\text{logit}(\mathbb{P}[Y_{i,j,l}(s) \mid X_{i,j}(s)]) = \beta_0(s) + X_{i,j}(s)\beta_1(s),$$

where $X_{i,j}(s) \in \mathbb{R}$ is a measurement of whisker activtiy at trial timepoint $s$ on trial $j$ for the animal that neuron $i$ was recorded from. We centered and scaled whisker activity for interpretability.

Because $\widehat{\beta}_1(s)$ is significantly positive throughout the entire trial (i.e. across the functional domain), suggests that the neuron–whisker association is not driven by whisker stimulation. Interestingly the magnitude of association is roughly twice as high with the AR1 structure. Both the intercept and the functional slope suggest that the whisker stimulation (occurring at timepoint 0 sec) has only a small influence on the association between neural activity and whisker activity. Since $X_{i,j}(s)$ is centered and scaled, the functional intercept can be interpreted as the mean log odds of neural activity at an average speed. This appears to be affected by the stimulation as the shape of the functional intercept appears to rapidly increase and then decrease after stimulation (timepoint 0 sec). The AR1 analysis, for example, took 13.5min, illustrating its speed for large datasets with functional covariates.

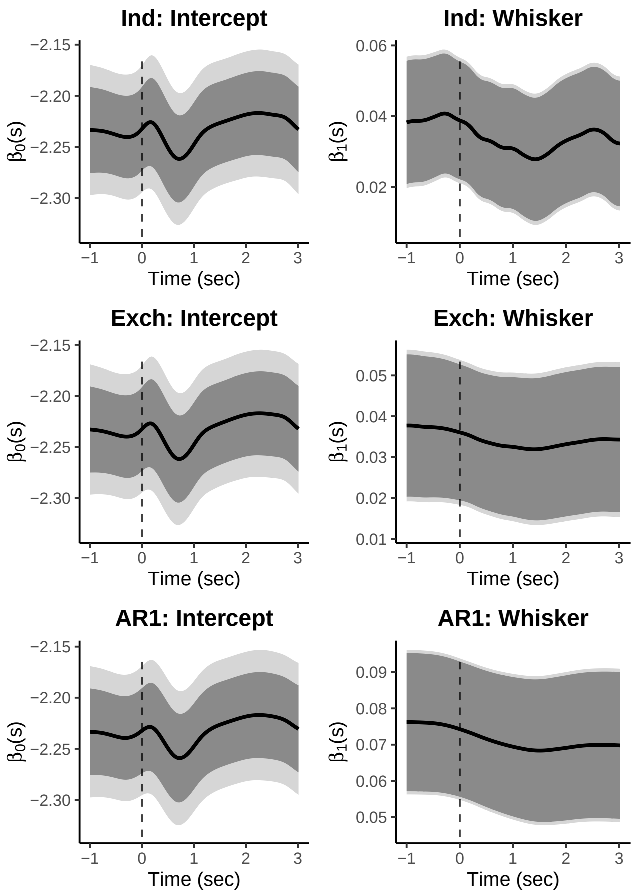


Figure 2: **Whisker–Neural activity association.** Functional coefficient estimates for Independent (Ind), Exchangeable (Exch), and Auto-regressive-1 (AR1) working correlation structures. Time relative to whisker stimulation.
